# N-Succinylated Canonical vs. Dehydropeptides: Contrasting Self-Assembly Pathways and Hydrogel Properties

**DOI:** 10.3390/gels12040299

**Published:** 2026-04-01

**Authors:** Teresa Pereira, André F. Carvalho, Filipe Teixeira, Andreia Patrícia Magalhães, David M. Pereira, Loic Hilliou, Antero Abrunhosa, Manuel Bañobre-López, Paula M. T. Ferreira, José A. Martins

**Affiliations:** 1 Center of Chemistry, University of Minho, 4710-057 Braga, Portugal; id10165@alunos.uminho.pt (T.P.); id9569@alunos.uminho.pt (A.F.C.); fteixeira@quimica.uminho.pt (F.T.); 2 Advanced (Magnetic) Theranostic Nanostructures Lab, International Iberian Nanotechnology Laboratory (INL), Av. Mestre José Veiga s/n, 4715-330 Braga, Portugal; andreia.magalhaes@inl.int (A.P.M.); manuel.banobre@inl.int (M.B.-L.); 3 REQUIMTE/LAQV, Laboratório de Farmacognosia, Departamento de Química, Faculdade de Farmácia, Universidade do Porto, R. Jorge Viterbo Ferreira, n 228, 4050-313 Porto, Portugal; dpereira@ff.up.pt; 4 Institute for Polymers and Composites, University of Minho, 4800-058 Guimarães, Portugal; loic@dep.uminho.pt; 5 Coimbra Institute for Biomedical Engineering and Translational Research (CIBIT)/Institute for Nuclear Sciences Applied to Health (ICNAS), University of Coimbra, 3000-548 Coimbra, Portugal; antero@pet.uc.pt

**Keywords:** N-succinyl (dehydro)peptides, self-assembly, hydrogels, proteolytic resistance, self-healing

## Abstract

Limited mechanical robustness and prompt proteolytic degradation preclude wider biomedical application of supramolecular peptide hydrogels. Low-molecular-weight dehydropeptides represent a promising class of hydrogelators, owing to their enhanced proteolytic stability, high self-assembly propensity, biocompatibility, and tunable rheological and drug-release properties. Herein, we prepared a small library of N-succinylated dehydrotripeptides (Suc-L-Xaa-L-Phe-Z-ΔPhe-OMe/-OH; Xaa = Phe or Val), together with the canonical analogs (Suc-L-Phe-L-Phe-L-Phe-OMe/-OH), to assess whether in addition to proteolytic resistance, dehydropeptides offer clear advantages over canonical peptides in terms of self-assembly, gelation efficacy, mechanical performance, and cargo release. Peptide self-assembly, hydrogel formation, and supramolecular organization were investigated by fluorescence and circular dichroism (CD) spectroscopy, molecular dynamic (MD) simulations, Thioflavin T hydrogel staining, ATR-FTIR spectroscopy, transmission electron microscopy (TEM), and rheological measurements. Drug-release performance was evaluated using methyl orange as a model cargo. Overall, the dehydropeptide-based hydrogels displayed enhanced gelation efficacy, improved mechanical properties, and sustained release profiles compared to canonical analogs. Spectroscopic analysis (CD and ATR-FTIR) and molecular dynamic simulations indicated that the dehydropeptides preferentially self-assemble into more ordered supramolecular fibrils, with extended β-sheet-like packing, whereas the canonical peptides predominantly populate more disordered backbone environments. Proteolysis assays with α-chymotrypsin revealed that both canonical and dehydropeptide methyl esters underwent chymotrypsin-catalyzed ester hydrolysis. Importantly, only the canonical dicarboxylic acid underwent further proteolytic degradation. The dehydropeptide dicarboxylic acids revealed fully resistant to proteolysis over extended time periods. These results demonstrate that the incorporation of dehydroamino acid into peptides enables control over supramolecular packing, nanofibrillar network architecture, rheology, and cargo release. This report raises the profile of relatively underexplored dehydropeptide-based soft materials as promising high-performance biomaterials for technological and biomedical applications.

## 1. Introduction

Chemical synthesis enabled the development of modern therapeutics, (nano)theranostic agents, and functional (bio)materials. Advances in covalent synthetic methodologies, allied with the wide availability of building blocks and automation, have expanded the accessible chemical space. Nonetheless, the stepwise construction of complex molecules remains time-consuming and resource-intensive [1,2]. In contrast, non-covalent synthesis allows for fast one-step formation of hierarchically organized structures (with emergent properties) through molecular self-assembly, defined here as spontaneous organization of molecules into nanostructures driven by reversible non-covalent interactions [3,4]. Self-assembly relies on dynamic networks of delocalized weak intermolecular interactions, including electrostatic forces and hydrogen bonding, van der Waals interactions, π–π stacking, and hydrophobic effects, endowing supramolecular materials with self-correction, programmability, and intrinsic responsiveness to external stimuli [3,4,5,6,7]. Protein folding, directed by the information encoded in the amino acid sequence, generates unique three-dimensional structures with a specific biological function, thanks to an extensive ensemble of non-covalent interactions [8]. Inspired by natural systems (proteins, nucleic acids, biomembranes) that rely upon self-assembly for emerging function, short peptides, particularly di- and tripeptides, have emerged as minimalist building blocks for the construction of supramolecular nanostructures and nanostructured soft (bio)materials [4,5,6,7]. Facile synthesis and automation potential, intrinsic biocompatibility, and chemical versatility have driven immense interest in peptide-based self-assembled materials [8,9,10,11,12]. Due to limited intramolecular contacts and high conformational entropy, low-molecular-weight peptides fail to adopt stable globular structures. Instead, short peptides usually undergo multistate hierarchical self-assembly pathways (monomer → oligomer → nucleus → fibril), forming higher-order supramolecular aggregates stabilized by intermolecular peptide–peptide and peptide–solvent interactions [5,13]. The (limited) chemical space of short peptides can be expanded through terminal and side-chain modifications, enabling controlled modulation of charge, hydrophobicity, and intermolecular interactions. Bulky aromatic N-capping groups promote molecular packing, enhanced nucleation, and direct anisotropic assembly pathways, due to reduced electrostatic repulsion, π–π stacking, and hydrophobic collapse. Peptide N-capping also directs nanoscale peptide alignment, controls β-sheet organization, induces hierarchical transitions, and can function as chemical or enzymatic switches for controlled assembly and disassembly [14,15,16]. Despite these advantages, peptide-based materials are inherently susceptible to proteolytic degradation in biological environments, limiting their in vivo stability and functional lifetime. Enhancing the proteolytic resistance of peptide materials is critical for applications in drug delivery, tissue engineering, and the development of theragnostic agents. The incorporation of D- and non-canonical amino acids (e.g., β-amino acids) into peptides and cyclization approaches are robust strategies to augment protease resistance. Proteolytic stability results from the disruption of molecular recognition, restricted backbone accessibility and stabilization of specific secondary structures [5,17,18,19,20]. Peptide-based hydrogels are the most successful result of supramolecular peptide self-assembly. Protease-resistant peptide hydrogels benefit from longer lifetimes and more predictable mechanical properties, which translates into improved in vivo performance [21,22,23]. Over the years, our research group has developed a variety of peptide-based gelators, incorporating the dehydroamino acids dehydroalanine (ΔAla), dehydroaminobutyric acid (ΔAbu), and dehydrophenylalanine (ΔPhe). Dehydropeptides combine enhanced proteolytic resistance with high self-assembly propensity and efficient gelation. C-deprotected N-capped dehydropeptide architectures demonstrated tunable gelation and rheological properties and excellent performance as nanocarriers for drug delivery applications [24,25,26,27,28]. We have also demonstrated that dehydropeptide stereochemistry (homochirality vs. heterochirality) can direct the self-assembly pathway, hydrogel morphology and rheological properties [29,30]. Our research group developed efficient synthetic methodologies for dehydropeptides, based on the dehydration of dipeptide blocks containing a C-terminal β-hydroxyamino acid (Ser, Thr or β-hydroxyphenylalanine). The requirement of an β-hydroxyamino acid and an additional dehydration step makes the synthesis of dehydropeptides more demanding than the synthesis of canonical peptides, which are readily accessible by solid-phase methods. In this context, it is legitimate to question whether, beyond proteolytic resistance, dehydropeptides display definite advantages that justify the additional synthetic effort. In this work, a small library of homochiral N-succinylated tripeptides, Suc-L-Xaa-L-Phe-ΔPhe-OMe/-OH (Xaa = Phe or Val), was synthesized alongside their canonical tripeptide analogs, Suc-L-Phe-L-Phe-L-Phe-OMe/-OH [30]. The self-assembly behavior, gelation and rheological properties and the drug delivery performance of the canonical and dehydropeptide-based hydrogels were directly compared. Furthermore, hydrolysis studies with chymotrypsin were conducted to assess the impact of dehydroamino acid incorporation on the proteolytic resistance [31].

## 2. Results and Discussion

### 2.1. Synthesis

**Scheme 1:**
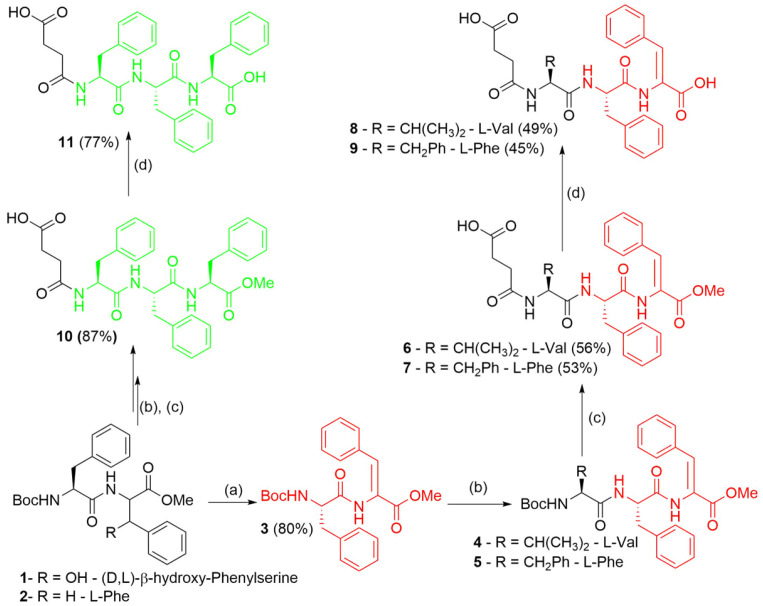
Synthetic route for N-succinylated (dehydro)tripeptides (Suc-L-Xaa-L-Phe-∆Phe-OMe/-OH; Suc = Succinic acid; Xaa = L-Phe, L-Val), featuring the L-Phe-∆Phe motif, and for the canonical amino acids analogs, Suc-L-Phe-L-Phe-L-Phe-OMe/-OH: (**a**) (i) Boc_2_O, DMAP, dry MeCN, (ii) TMG; (**b**) Boc-AA-OH, HBTU, Et_3_N, MeCN; (**c**) (i) TFA, (ii) succinic anhydride, pyridine, N_2_ atmosphere; and (**d**) (i) NaOH (1 M), 1,4-dioxane, (ii) HCl (1 M).

**Figure 1:**
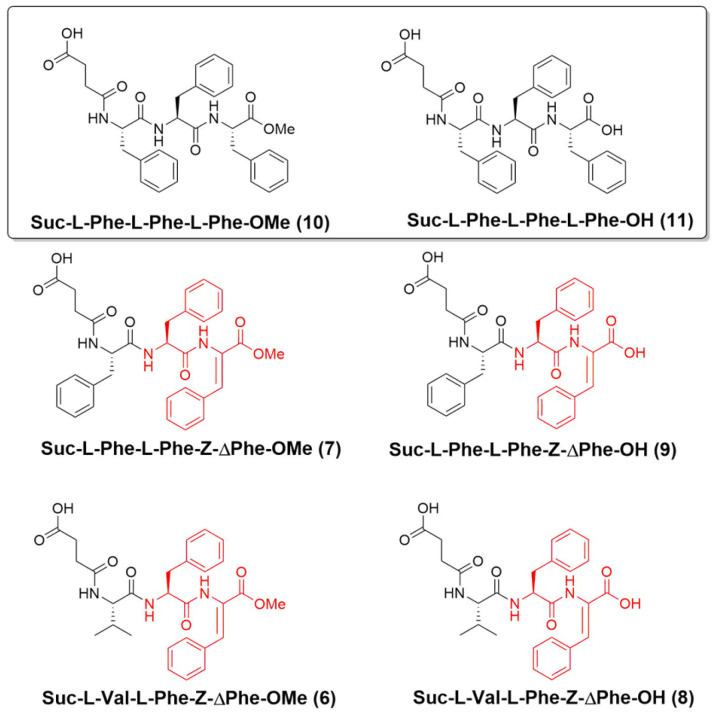
Focused library of N-succinylated (dehydro)tripeptides, featuring the Phe-∆Phe motif and their canonical amino acids analogs, Suc-L-Phe-L-Phe-L-Phe-OMe/-OH, studied in this work.

A small library of N-succinylated dehydrotripeptides bearing a C-terminal L-Phe-ΔPhe motif and either L-Phe or L-Val at the N-terminus was synthesized using a solution-phase Boc strategy [30]. The corresponding canonical amino acid analogs, Suc-L-Phe-L-Phe-L-Phe-OMe/-OH, were also prepared for comparison purposes (Scheme 1 and Figure 1).

The Boc-protected dehydrodipeptide block **3**, featuring the L-Phe-Z-ΔPhe motif, was used as a modular precursor for the diversification of the peptide library (Scheme 1). Boc-protected dehydrotripeptides **4** and **5** were synthesized by TFA deprotection of dehydropeptide **3**, followed by a chain extension with Boc-protected L-Val or L-Phe under standard HBTU/TEA coupling conditions. N-terminal deprotection of dehydrotripeptides **4** and **5**, followed by acylation with succinic anhydride, afforded N-succinylated dehydrotripeptides **6** and **7** as methyl esters. Conversion to dicarboxylic acids **8** and **9** was achieved by alkaline hydrolysis. All compounds were obtained in overall good yields through aqueous workup procedures and ether precipitation. The dehydration of dipeptide Boc-L-Phe-L,D-β-hydroxyphenylalanine methyl ester **1** represents the key transformation in the synthesis of the dehydropeptides [30,32]. The N-succinylated canonical analogs **10**/**11** of dehydropeptides **7**/**9** were prepared by an analogous synthetic route, starting from easily accessible Boc-protected dipeptide L-Phe-L-Phe-OMe (**2**).

The N-succinyl peptide molecular architecture described herein has distinct advantages compared to conventional N-acetylation or N-capping with aromatic groups. Peptide N-succinylation generates a polarity reversal compared to N-capped C-deprotected peptides. The N-succinyl group not only adds solubility, but is also a molecular switch for pH drop-triggered gelation of C-protected peptides. The saponification of methyl ester peptides gives direct access to the corresponding dicarboxylic acids. Distinct molecular properties are likely to translate into different self-assembly pathways for the methyl ester and for the dicarboxylic acid hydrogelators. Selective methyl ester chemical or enzymatic transformations can potentially be deployed for tuning the properties of the hydrogelators/hydrogels [30].

The (dehydro)peptides were characterized by ^1^H-NMR spectroscopy, with special emphasis on the amide region, to get insight into the conformation of the peptide backbone and putative participation of the amide (NH) protons on hydrogen bonding networks. Three distinct signals, assigned to NH amide protons, were identified in the amide region (Table 1).

**Figure 2:**
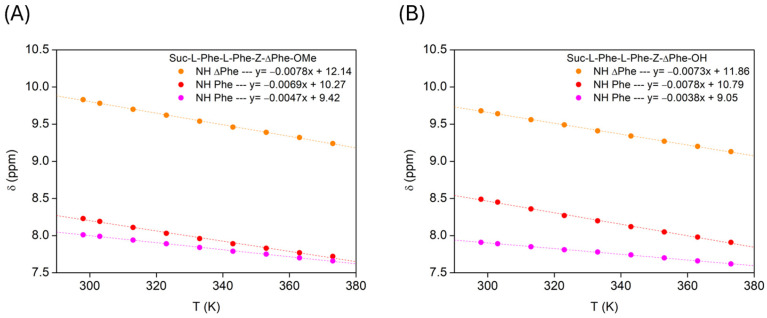
Variable-temperature ^1^H NMR studies (400 MHz; DMSO-d_6_) of the amide (NH) protons’ chemical shifts, illustrated here for dehydropeptides (**A**) Suc-L-Phe-L-Phe-Z-∆Phe-OMe (**7**) and (**B**) Suc-L-Phe-L-Phe-Z-∆Phe-OH (**9**). The full study can be found in the Supplementary Information as Figure S1.

The signal attributed to the amide proton of the ΔPhe residue of the dehydropeptides appears at a significantly higher chemical shift than the amide signals of the canonical amino acid residues, due to conjugation of the *C*α = *C*β double bond with the amide π–π system. Owing to the inductive effect of the succinyl N-capping group, the signal attributed to the N-terminal amide appears at a higher chemical shift than the signal attributed to the internal amide. For the canonical peptides (Suc-L-Phe-L-Phe-L-Phe-OMe/-OH (**10**/**11**), the chemical shift in the internal and C-terminal amides is nearly identical, reflecting high structural symmetry. The N-terminal amide exhibits a distinctively higher chemical shift, due to the electron withdrawing effect of the succinyl group. Variable-temperature (298–373 K) ^1^H-NMR studies were performed to obtain insight into the conformational stability and putative self-assembly of the (dehydro)peptides (Figure 2 and Figure S1) [33].

The temperature coefficient (Δδ/ΔΤ; ppb/K) for the NH amides was calculated from the temperature dependence of the NH amides’ chemical shift (Figure 2, Table S1). Solvent-exposed amide protons usually display temperature coefficients above −5 ppb/K. Values below −4 ppb/K strongly suggest solvent-protected amides, which are likely involved in intra- or intermolecular hydrogen bonds. The amide signals of the canonical peptides Suc-L-Phe-L-Phe-L-Phe-OMe/-OH (**10**/**11**) show temperature coefficients above the threshold for intra- or intermolecular hydrogen bonding, suggesting a flexible random-coil-like structure. The NHΔPhe (C-terminal) amide of the dehydropeptides, both ester and dicarboxylic acids, seems fully exposed to the solvent. The N-terminal and the internal amide bonds seem more shielded from the solvent. Interestingly, the temperature coefficient for the internal amide of the dicarboxylic acid Suc-L-Phe-L-Phe-Z-ΔPhe-OH (**9**), −3.8 ppb/K, indicates that this amide is protected from the solvent and is likely participating in a stable intra- or intermolecular hydrogen bond. The lower steric bulk of the Val side chain is likely to relieve steric hindrance, which potentially limits π–π stacking of the aromatic rings. In fact, the MD simulations (see below) revealed that the π–π stacking interaction could be detected only for dehydropeptide Suc-L-Val-L-Phe-Z-∆Phe-OH (**8**), which formed the most compact aggregates. The substitution Phe–>Val seems to trigger a different self-assembly pathway.

### 2.2. Peptide Self-Assembly

#### 2.2.1. Critical Aggregation Concentration

**Figure 3:**
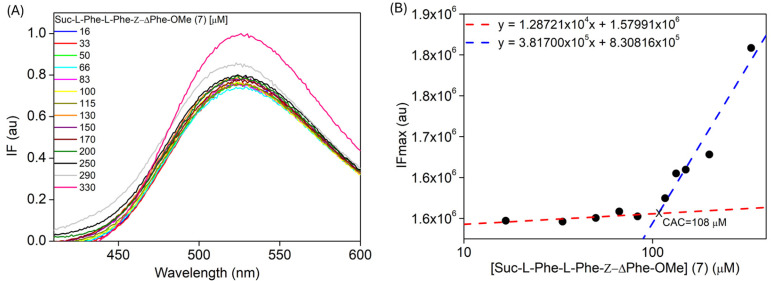
Experimental methodology for determination of CAC values, illustrated here for peptide **7**: (**A**) steady-state fluorescence spectra (*λ*_exc_ = 370 nm) for dehydropeptide Suc-L-Phe-L-Phe-Z-ΔPhe-OMe (**7**) in the concentration range 330 to 16 μM in the presence of added ANS (25 μM); (**B**) semilogarithmic graphical representation of the concentration dependence of the fluorescence maximum emission intensity of the ANS probe (*λ*_exc_ = 370 nm). The full set is presented in the Supplementary Information as Figure S2.

The parameter critical aggregation concentration (CAC), typically in the micromolar concentration range, signals the onset of concentration-dependent peptide self-assembly into higher-order supramolecular aggregates, e.g., micelles, protofibrils, or nanofibers. The descriptor of molecular hydrophobicity and lipophilicity cLogP (calculated LogP) measures the propensity of molecules to undergo hydrophobic collapse-driven self-assembly [34]. Owing to the absence of intrinsic sensitive chromophores in the (dehydro)peptides, the polarity sensor 8-anilinonaphthalene-1-sulfonic acid (ANS) was employed as an extrinsic fluorescence probe for CAC determination. Hydrophobic collapse-driven peptide aggregation generates nonpolar microdomains that bind ANS, causing an abrupt increase in fluorescence intensity, which is typically accompanied by a blue shift in the emission maximum [35,36]. CAC values were determined from the peptide concentration’s dependence on the maximum ANS fluorescence emission (*λ*_exc_ = 370 nm) (Figure 3, Table 2).

The CAC values were interpreted as the intersection point of the straight lines representing the monomeric and aggregate regimens in the semilogarithmic graphic representation (Figure 3B and Table 2).

The CAC values for the (dehydro)peptides are of the same order of magnitude, between 100 and 155 µM, but significantly lower than their peptide solubility (Table 2 and Table S2). As a general trend, higher hydrophobicity (c*L*ogP values) seems to translate into lower CAC values, i.e., higher aggregation propensity. The canonical amino acids peptide couple **10**/**11**, exhibiting the lowest hydrophobicity, displays the highest CAC values. A direct comparison with the analogous dehydropeptides **7**/**9** highlights the self-assembly propensity-enhancing properties of the dehydroamino acid. For the methyl ester/dicarboxylic acid peptide couples, the difference in hydrophobicity between the ester and the carboxylic acid seems to not translate into substantial differences in self-assembly propensity. Dehydropeptide **8** is a truncated dehydro analog of the Lys-Leu-Val-Phe-Phe (KLVFF) motif of the β-amyloid Alzheimer’s peptide. The fully deprotected and the C-amidated analogs of the Aβ-peptide display an exceptionally high self-assembly propensity (CAC = 0.01 wt%; 1.57 µM for the amidated peptide), much higher than that displayed by peptide **8** [36,37]. In line with this trend, dicarboxylic acid Suc-L-Val-L-Phe-Z-∆Phe-OH (**8**), despite displaying the lowest hydrophobicity in the dehydropeptides’ series, displays the highest self-assembly propensity. The self-assembly of dehydropeptides Suc-L-Phe-L-Phe-Z-∆Phe-OMe/-OH (**7/9**) is likely driven by aromatic π–π stacking interactions that favor extended (β-sheet-like) peptide backbone conformations [38,39,40]. The substitution Phe–>Val may not only relieve putative steric constraints to the supramolecular packing of aromatic rings, but can also add van der Waals and hydrophobic interactions that seem to trigger a distinct self-assembly pathway, as seen in the VT NMR experiments (Figure 2). Moreover, the MD simulation’s observation that dehydropeptide Suc-L-Val-L-Phe-Z-∆Phe-OH (**8**) self-assembles into more compact aggregates than peptide Suc-L-Phe-L-Phe-Z-∆Phe-OH (**9**) suggests higher self-assembly propensity. Direct comparison with peptide Suc-L-Phe-L-Phe-Z-∆Phe-OH (**9**) highlights the importance of the alkyl side chain of the Val residue for self-assembly.

#### 2.2.2. Circular Dichroism

**Figure 4:**
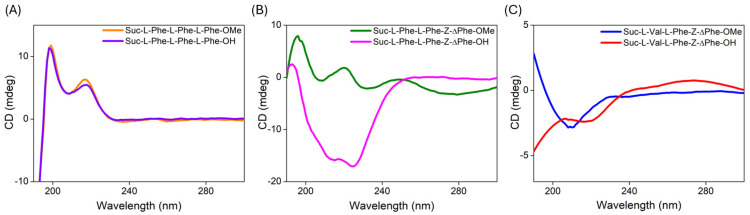
Circular dichroism spectra for hydrogelator solutions (0.01 wt%): (**A**) Suc-L-Phe-L-Phe-L-Phe-OMe/-OH (**10**/**11**); (**B**) Suc-L-Phe-L-Phe-Z-∆Phe-OMe/-OH (**7**/**9**); and (**C**) Suc-L-Val-L-Phe-Z-∆Phe-OMe/-OH (**6**/**8**). The corresponding UV–Vis and HT spectra are shown in the Supplementary Information (Figures S3 and S4, respectively).

Circular dichroism (CD) spectroscopy was deployed to gain insight into the conformation of the peptide backbone in the self-assembled peptide nanostructures and to probe chiral environments generated by peptide self-assembly (Figure 4).

The CD spectra of the canonical peptides Suc-L-Phe-L-Phe-L-Phe-OMe/-OH (**10**/**11**) suggest that peptide self-assembly is driven by aromatic interactions, instead of repetitive backbone conformations. The strong positive band at around 200–205 nm, together with the broad shoulder around 218–220 nm, can be assigned to the aromatic exciton coupling (π–π* transitions) resulting from the chiral supramolecular arrangements of aromatic rings via π–π stacking interactions [41]. The strong negative band at around 220 nm and the weak positive band at around 195 nm in the CD spectra of the dehydropeptides Suc-L-Phe-L-Phe-Z-∆Phe-OMe/-OH (**7**/**9**) can be attributed to extended (β sheet-type) peptide backbone conformations. The CD spectrum of the dicarboxylic acid Suc-L-Phe-L-Phe-Z-∆Phe-OH (**9**) displays spectral features that are much stronger than its methyl ester counterpart (**7**), suggesting a larger fraction of extended peptide backbone conformations. The Val-containing dehydropeptides Suc-L-Val-L-Phe-Z-∆Phe-OMe/-OH (**6**/**8**) display similar, featureless spectra, showing a broad negative band around 210 nm. N-terminal substitution Phe–>Val seems to result in different peptide backbone conformations. Owing to the instrument’s limitations, the CD spectra were acquired at concentrations of 0.01 wt%, well below the peptides’ critical gelation concentrations (0.2 wt%). The CD spectra are not truly representative of the gel state, but instead represent a pre-gelation state.

#### 2.2.3. Molecular Dynamics Simulations

**Figure 5:**
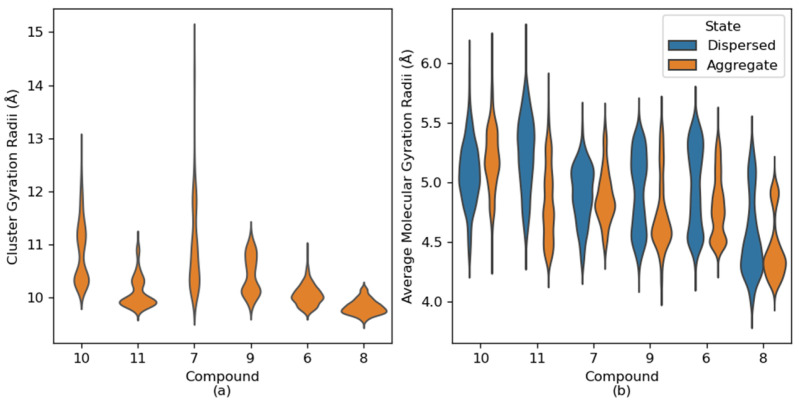
Distribution of the gyration radii (Å) for the (dehydro)peptide aggregates (**a**) and for the dehydropeptide molecules in the D and A states (**b**).

**Figure 6:**
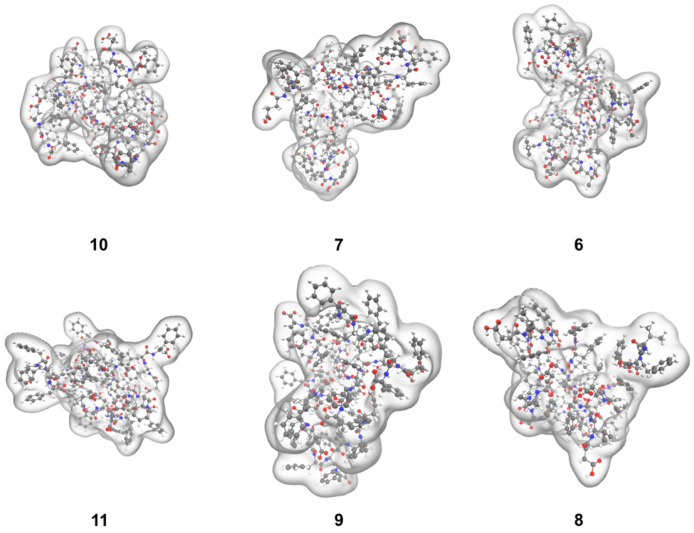
Cluster structures obtained by MD simulation for the aggregate state of the dehydropeptides: Suc-L-Phe-L-Phe-L-Phe-OMe/-OH (**10**/**11**); Suc-L-Phe-L-Phe-Z-∆Phe-OMe/-OH (**7**/**9**) and Suc-L-Val-L-Phe-Z-∆Phe-OMe/-OH (**6**/**8**). (Carbon atoms are represented as grey spheres; Oxygen atoms in red; Nitrogen atoms in blue).

Molecular dynamics (MD) simulations were carried out to gain insight into the self-assembly pathway of the (dehydro)peptides and the supramolecular packing of the aggregates. Two states were considered: in the dispersed state (D), each molecule is fully solvated, whereas in the aggregated state (A), the molecules are condensed into a single aggregate. The distribution of intermolecular distances between the geometric center of the (dehydro)peptide molecule (d_C_) in the production run reflects the initial configuration of each state (Figure S5). Both the canonical and dehydropeptides formed tightly packed aggregates, as indicated by the small amplitude of the calculated d_C_ values for the A state. Interestingly, dehydropeptide Suc-L-Val-L-Phe-Z-∆Phe-OH (**8**) formed the most compact aggregates. In the D state, a transient dimer formation (d_C_ < 10 Å) was observed for some systems, especially for dehydropeptide Suc-L-Phe-L-Phe-Z-∆Phe-OH (**9**). The reduction in the solvent accessible surface area (SASA) resulting from aggregates’ formation was calculated as a potential self-assembly propensity metric (Table S3). With the exception of peptide Suc-L-Val-L-Phe-Z-∆Phe-OMe (**6**), the SASA reduction upon aggregation was similar for all peptides. The gyration radius of the aggregates provided further insights into the structure (size and shape) of the clusters (Figure 5 and Figure 6).

The large value of the gyration radius observed for compound Suc-L-Phe-L-Phe-Z-∆Phe-OMe (**7**) reflects the detachment of one molecule from the cluster. In general, the methyl ester dehydropeptides show a propensity for formation of more oblong, less compact aggregates than their dicarboxylic acid counterparts, resulting in aggregates with significantly higher gyration radii. Remarkably, dehydropeptides Suc-L-Val-L-Phe-Z-∆Phe-OMe/-OH (**6**/**8**), featuring a N-terminal Val residue, exhibit rounder aggregates with a more stable shape (Figure 5a and Figure 6).

**Figure 7:**
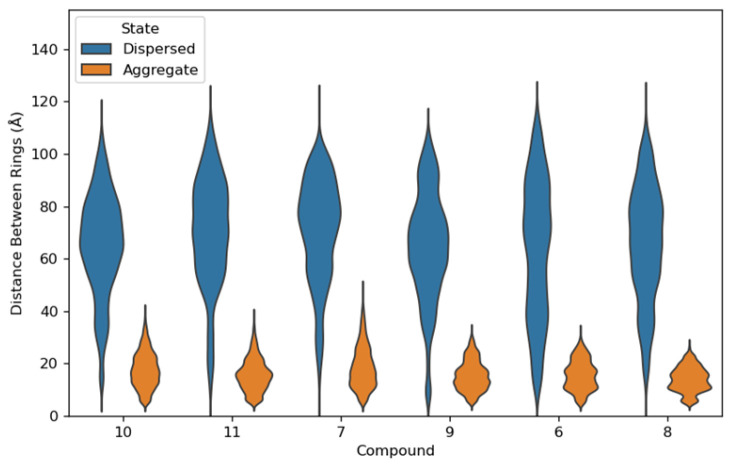
Distribution of intermolecular distances between the center of the aromatic systems for the (dehydro)peptides, both in the aggregated and dispersed states.

The distribution of the gyration radii was also studied for the individual dehydropetide molecules, both in the dispersed and aggregate states, to get insight into their conformational space (Figure 5b). The individual molecular gyration radii of all compounds follow approximately the same distribution in the aggregated and dispersed states, with some concentration around the modal peak in the aggregated state. The canonical peptides Suc-L-Phe-L-Phe-L-Phe-OMe/-OH (**10**/**11**) display extended, very flexible conformations, both in the D and A states, whereas the dehydropeptides tend to adopt less extended conformations. Interestingly, for dehydropeptide Suc-L-Phe-L-Phe-Z-∆Phe-OMe (**7**), both states display a mono-modal distribution, with the modal peak in the dispersed state at 5.4 Å, and the one for the aggregate at 4.7 Å, indicating that the individual molecules display similar conformation in both states. The distribution of intermolecular distances between the centers of the aromatic systems was also sampled during the production runs, in order to gain insight into the contribution of intermolecular π–π stacking interactions to molecular aggregation (Figure 7).

In general, as indicated by the modal peak at around 60 Å and as also seen in Figure S6, π–π stacking is negligible for the dispersed state of all peptides. In contrast, the inter-ring distances are greatly reduced in the aggregate state, with the modal peaks between 10 and 15 Å reflecting the spatial proximity of the molecules. Only dehydropeptide Suc-L-Val-L-Phe-Z-∆Phe-OH (**8**) exhibits a clear minor modal peak below 5 Å, suggesting the presence of a stable set of π–π stacked rings, likely in a sandwich conformation. Importantly, all compounds show a modal peak at around 8.0 Å, which is consistent with T-shaped intermolecular aromatic interactions [42]. The formation of inter- and intramolecular, peptide–peptide and peptide–water hydrogen bonds in the D and A states, was also acquired during the aggregation trajectories (Figures S6 and S7). Peptide aggregation results in a significant reduction in the number of intermolecular peptide–water hydrogen bonds, which is only partially compensated by the formation of peptide–peptide hydrogen bonds. A clear increase of five or more peptide–peptide hydrogen bonds (per 10 peptide molecules) following peptide aggregation is only observed for dehydropeptides Suc-L-Phe-L-Phe-Z-∆Phe-OMe (**7**) and Suc-L-Val-L-Phe-Z-∆Phe-OMe/-OH (**6**/**8**) and for the canonical dicarboxylic acid Suc-L-Phe-L-Phe-L-Phe-OH (**11**). The remaining compounds retain approximately the same average number of peptide–peptide hydrogen bonds in both states, suggesting that the geometry of these molecules is governed by strong intramolecular hydrogen bonds. Moreover, the results presented in Figure 6 and Figure 7 further confirm that the formation of molecular dimers in the dispersed phase is likely mediated by the approximation of aromatic moieties, rather than the formation of intermolecular hydrogen bonds. Overall, the MD simulations indicate that peptide aggregation is dominated by intermolecular π–π stacking interactions.

### 2.3. Hydrogel Studies

#### 2.3.1. Preparation of Hydrogels

**Figure 8:**
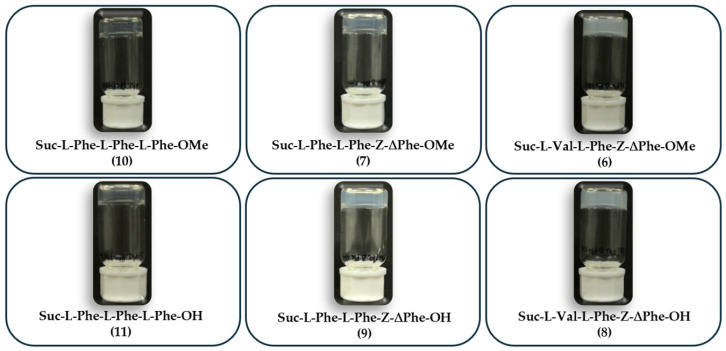
Demonstration of formation of self-supporting hydrogels by the inverted tube test.

N-succinylation was devised as a strategy to endow C-protected hydrophobic peptides with aqueous solubility and to act as a molecular trigger for supramolecular gelation via pH-drop methodologies. The N-succinylated methyl ester peptides exhibit a polarity inversion, compared to C-deprotected peptides N-capped with non-ionizable aromatic moieties. Electrostatic effects may trigger different self-assembly pathways for the methyl ester and the dicarboxylic acid peptides [30]. The peptides were solubilized in water by adjusting suspensions to pH 9–10 with NaOH. Gelation was triggered by homogeneous solution acidification via the hydrolysis of glucono-δ-lactone (GDL) [43]. Hydrogel formation was confirmed by tube inversion (Figure 8 and Table 3).

Interestingly, whilst the methyl ester peptides **6**, **7** and **10** required only a mass equivalent of GDL for gelation, the dicarboxylic acid peptides **8**, **9** and **11** required at least 2 mass equivalents. Gelation seems to be initiated by a pH-triggered hydrophobic collapse. The final pH of the hydrogels can be tuned into the physiological range by adjusting the amount of GDL. Whereas the canonical peptides **10** and **11** afforded gels at 0.3 wt% concentration, the dehydropeptide couples **6**/**8** and **7**/**9** afforded visually strong hydrogels at 0.2 wt%, in line with the lower CAC values measured for the dehydropeptides. Overall, the dehydropeptides are more efficacious gelators than their canonical analogs.

#### 2.3.2. Thioflavin-Stained Hydrogels

**Figure 9:**
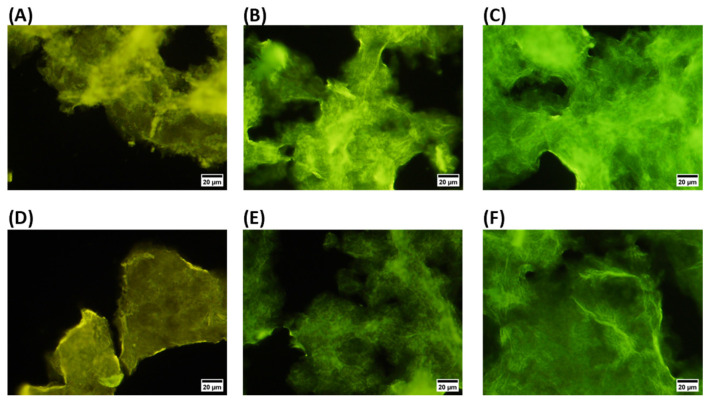
Fluorescence microscopy images of dehydropeptides hydrogels (0.4 wt%) labeled with thioflavin T: (**A**) Suc-L-Phe-L-Phe-L-Phe-OMe (**10**); (**B**) Suc-L-Phe-L-Phe-Z-∆Phe-OMe (**7**); (**C**) Suc-L-Val-L-Phe-Z-∆Phe-OMe (**6**); (**D**) Suc-L-Phe-L-Phe-L-Phe-OH (**11**); (**E**) Suc-L-Phe-L-Phe-Z-∆Phe-OH (**9**); and (**F**) Suc-L-Val-L-Phe-Z-∆Phe-OH (**8**).

Thioflavin T (ThT) is a sensitive reporter for the detection of amyloid-type peptide structures. Upon binding to extended β-sheet stacks, fibrillar and protofibril assemblies as well as supramolecular β-sheet hydrogel networks, ThT exhibits a strong increase in fluorescence emission [44]. Fluorescence microscopy images of ThT-stained hydrogels (0.4 wt%) were acquired to gain insight into the organization of the self-assembled fibrillar networks in the (dehydro)peptide-based hydrogels (Figure 9).

The dehydropeptide-based hydrogels **7**/**9** and **6**/**8** show interconnected fibrous domains exhibiting intense green fluorescence, consistent with the supramolecular fibers featuring extended β-sheet-like ordering that are characteristic of amyloid-like fibrillar networks. The canonical peptide hydrogels **10**/**11** show mostly yellowish fluorescence, suggesting a lower degree of extended peptide backbone organization [41,45]. The Suc-L-Val-L-Phe-Z-∆Phe-OMe/-OH hydrogels (**6**/**8**) seem to display the highest content of extended β-sheet-like structuring. Moreover, UV–Vis binding assays to Congo red showed that the dehydropeptide gels induced a significant increase in the intensity of the Congo red absorption band accompanied by broadening and red shift, which was consistent with the binding to ordered β-sheet-like structures. In contrast, the canonical hydrogels induced a decrease in the intensity of the Congo red absorption band, accompanied by a small red shift, indicating binding to less ordered structures (Figure S8). Together, the ThT and Congo red binding assays suggest that the dehydropeptide gels display amyloid-like fibrillar networks [36,37]. These results are in agreement with the CD study, which also suggests a higher degree of ordered extended β-sheet-like backbone conformations for the dehydropetides.

#### 2.3.3. Transmission Electron Microscopy (TEM) Studies

**Figure 10:**
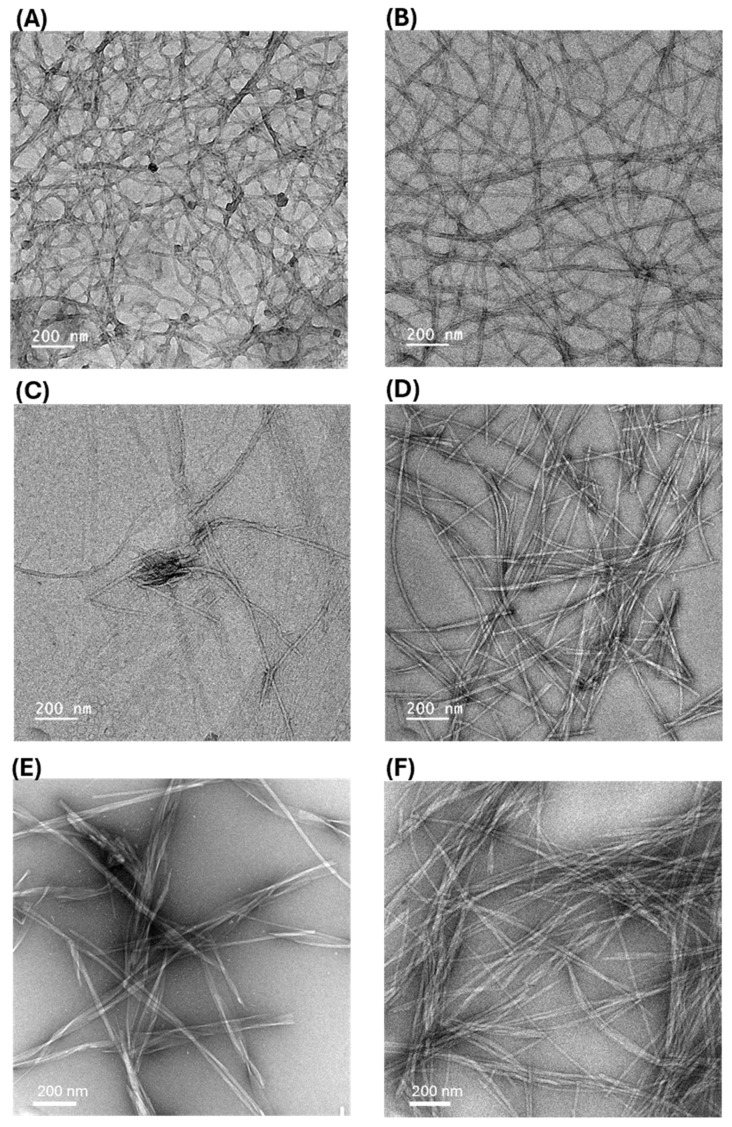
TEM images of (dehydro)peptide hydrogels (0.4 wt%): (**A**) Suc-L-Phe-L-Phe-L-Phe-OMe (**10**); (**B**) Suc-L-Phe-L-Phe-L-Phe-OH (**11**); (**C**) Suc-L-Phe-L-Phe-Z-∆Phe-OMe (**7**); (**D**) Suc-L-Phe-L-Phe-Z-∆Phe-OH (**9**); (**E**) Suc-L-Val-L-Phe-Z-∆Phe-OMe (**6**); and (**F**) Suc-L-Val-L-Phe-Z-∆Phe-OH (**8**).

Hydrogels are macroscopic materials that are hierarchically organized at the micro- and nanoscale. The micro- and nanostructure of the peptide hydrogels was investigated by transmission electron microscopy (TEM). Hydrogel samples (0.4 wt%) were deposited onto copper mesh TEM grids and stained with a uranyl-free stain (Figure 10).

The TEM images reveal that all hydrogels consist of fibrillar networks that are characteristic of self-assembled peptide hydrogels. Significant differences in fiber length and thickness, network density, and the degree of entanglement and fiber bundling can be observed among the different hydrogels. The canonical peptide hydrogels (Figure 10A,B) exhibit a higher network density and higher degree of entanglement than the dehydropeptide-based hydrogels, which display some extent of fiber bundling (Figure 10C–F). Notably, the dehydropeptide-based dicarboxylic acid hydrogels form longer and thinner fibers and show higher network density and entanglement than their methyl ester counterparts. In addition, these hydrogels show some extent of lateral fibril association. The methyl ester hydrogel bearing the N-terminal Val residue (**6**) displays short and thick coiled-coil-like structures that seem to result from parallel fiber association.

#### 2.3.4. Fourier Transform Infrared Red (FTIR) Studies

**Figure 11:**
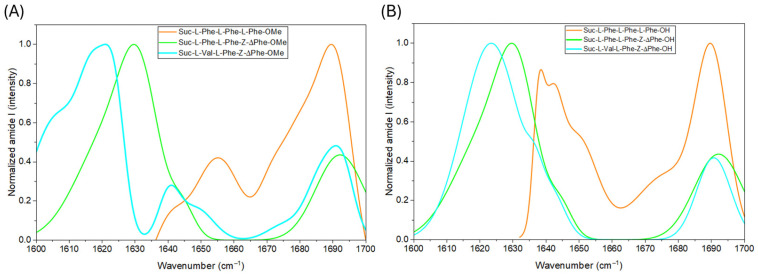
Comparison of the amide I region (1600–1700 cm^−1^) of the ATR FTIR spectra of canonical and dehydropeptides in their methyl ester (**A**) and dicarboxylic acid (**B**) forms. Spectra were smoothed and normalized to highlight differences in band shape.

Low-molecular-weight peptides, instead of adopting compact folded structures, self-assemble into supramolecular networks stabilized by intermolecular noncovalent interactions. The Amide I region (1600–1700 cm^−1^) of the (dehydro)peptide hydrogels (0.4 wt%, in D_2_O) was analyzed by ATR FTIR spectroscopy. Bands at 1550–1610 cm^−1^ and 1400–1450 cm^−1^ were assigned to asymmetric and symmetric carboxylate stretching modes, respectively, indicating partial ionization of the carboxylic acid groups at the hydrogel pH (~5–6) [46]. Spectral deconvolution of the Amide I region was performed to compare the peptide backbone hydrogen-bonding patterns within the hydrogels’ fibers. Bands near ~1690 cm^−1^ were assigned to constrained or turn-like backbone conformations and to the high-frequency component of antiparallel β-sheets; those around ~1655 cm^−1^ can be attributed to disordered random coil-like backbone environments and bands near ~1615–1630 cm^−1^ can be assigned to ordered extended supramolecular β-sheet-like packing. Together, bands at ca. 1615–1630 and 1690 cm^−1^ are a signature of antiparallel β-sheet conformations [47] (Figure 11 and Figure S9).

The deconvolution of the vibrational spectra of the canonical ester and dicarboxylic acid peptides Suc-L-Phe-L-Phe-L-Phe-OMe (**10**) are dominated by three component bands centered at 1650, 1680 and 1690 cm^−1^, which are indicative of predominantly disordered random coil-like peptide backbone environments. In contrast, the spectra of the dehydropeptide hydrogels Suc-L-Phe-L-Phe-Z-∆Phe-OMe/-OH (**7**/**9**) and Suc-L-Val-L-Phe-Z-∆Phe-OMe/-OH (**6**/**8**) show a noticeable reduction in the bands at a higher frequency (1650, 1680 and 1690 cm^−1^) and the appearance of intense low-frequency bands in the ~1630–1615 cm^−1^ region, which is consistent with a higher degree of supramolecular ordering of the peptide backbone in an extended (anti-parallel) β-sheet-like supramolecular packing (Table S4) [48,49]. Interestingly, compared to their ester counterparts, the dicarboxylic acid hydrogels showed an increased content of extended β-sheet-like arrangements, likely promoted by additional hydrogen-bonding and electrostatic interactions. In the dehydropeptides, the substitution of Phe–>Val results in a shift in the low frequency band at around 1615–1630 cm^−1^, presumably due to enhanced hydrophobic interactions. These results are consistent with the ThT staining experiments, which also indicated a higher content of β-sheet-like supramolecular arrangements for the dehydropeptides. Overall, the amide I signatures demonstrated that the dehydropeptide-based hydrogels formed more ordered and tightly packed β-sheet-like supramolecular networks than their canonical analogs. The FTIR analysis indicates that the conformational preferences inferred by CD spectroscopy for the (dehydro)peptides in the pre-gelation state are preserved (and most likely amplified) in the gel state.

#### 2.3.5. Rheological Studies

**Figure 12:**
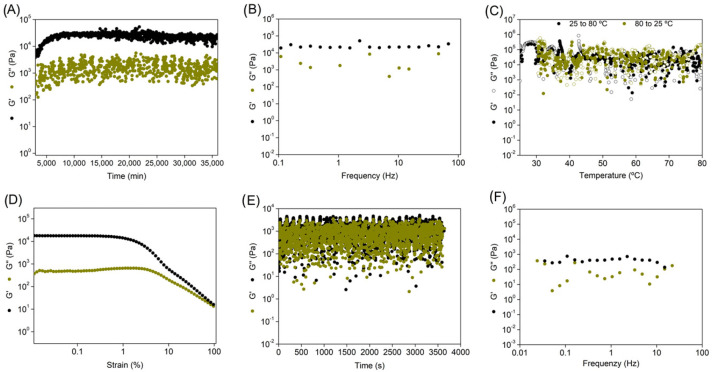
Experimental protocol implemented for characterization of the thermal–mechanical healing properties of (dehydro)peptide hydrogels, illustrated here for dehydropeptide **7**: (**A**) gelation kinetics; (**B**) mechanical spectrum, frequency sweep, for equilibrated gels; (**C**) temperature cycling; (**D**) strain sweep on rested gels after temperature cycling; (**E**) hydrogel reformation kinetics after mechanical breakdown; and (**F**) mechanical spectrum, frequency sweep, for reformed hydrogels. The full rheological characterization can be found in the Supporting Information (Figure S10).

**Figure 13:**
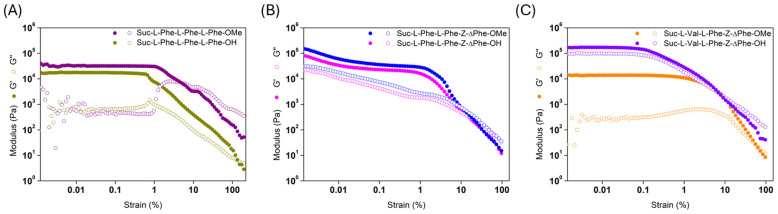
Strain sweep spectra for the (dehydro)peptide hydrogels. (**A**) **10/11**; (**B**) **7/9** and (**C**) **6/8** after thermal cycling.

Rheological measurements were performed at a 0.4 wt% hydrogelator concentration to evaluate the gelation kinetics, elastic properties, thermal sensitivity, and self-healing behavior of the hydrogels. The gelation kinetics was monitored over 10 h (Figure 12A). The elastic modulus of equilibrated gels (*G*′*_i_*) was obtained from frequency sweep measurements (Figure 12B). Thermal responsiveness was assessed by a heating–cooling cycle from 25–>80 °C (1 °C·min^−1^), followed by an equilibration period of 2 h at 25 °C. After thermal recovery, the mechanical stability of the hydrogels was evaluated by strain sweep experiments. The critical strain (*γ*_*c*_), defined as the strain at which *G*′ = *G*″, corresponding to gel network rupture and fluidization, was determined (Figure 12D and Figure 13). Structural recovery following gel break-up was monitored for 1 h (Figure 12E). The elastic modulus of the healed gel (*G*′*_r_*) was determined from frequency sweep measurements (Figure 12F). The hydrogels’ thermal healing efficiency was quantified as *TH* = *G*′*_h_*/*G*′*_i_* and the mechanical healing was measured as *MH* = *G*′*_r_*/*G*′*_h_*. A thermal–mechanical healing parameter, *TMH* = *G*′*_r_*/*G*′*_i_*, was also calculated to assess the cumulative effect of thermal and mechanical stresses on the elastic properties of the hydrogels [50,51].

All hydrogelators formed gels within 10 h. The storage modulus (*G*′*_i_*) was approximately one order of magnitude higher than the loss modulus (*G*″), confirming the formation of supramolecular hydrogels. With the exception of the canonical Suc-L-Phe-L-Phe-L-Phe-OH (**11**) and the dehydropeptide Suc-L-Val-L-Phe-Z-∆Phe-OMe (**6**) gels, the N-succinylated tripeptides formed remarkably strong gels [30]. Amongst the methyl ester-based gels, the canonical hydrogel Suc-L-Phe-L-Phe-L-Phe-OMe (**10**) and its dehydro analog Suc-L-Phe-L-Phe-Z-ΔPhe-OMe (**7**) displayed high elastic moduli (G’~250 kPa), whereas the gel Suc-L-Val-L-Phe-Z-ΔPhe-OMe (**6**) formed a significantly weaker gel (G’~20 kPa). This result is in accordance with the TEM images: while the Suc-L-Val-L-Phe-Z-ΔPhe-OMe (**6**) gel shows a low density fibrillar network, made of short and thick coiled-coil-like fibers, the canonical ester shows a high-density, highly entangled network. C-terminal methyl ester deprotection has contrasting effects on the elasticity of the resulting dicarboxylic acid hydrogels. A substantial decrease in *G*′ is observed for the canonical peptide (**11**), while a pronounced increase in elasticity is measured for the Val-containing hydrogel (**8**). After the thermal cycle, the recovery ability of the mechanically broken hydrogels was evaluated using the mechanical healing parameter (*MH* = *G*′*_r_/G*′*_h_;* Table 4). All hydrogels recovered their viscoelastic properties, although to different extents (18–71%). Hydrogels **7** and **10** showed the highest recovery degree (55% and 71%, respectively). With the exception of the methyl ester-derived hydrogels **7** and **10**, the remaining hydrogels showed significant thermal healing (80–150%). The mechanical strength, thermal stability and mechanical healing of the hydrogels can be correlated with their network architecture—namely, fiber thickness, network density, and the extent of entanglement and bundling, as observed in the TEM images (Figure 10). FTIR analysis indicates that the canonical peptides in the fibrillar networks of gels **10** and **11** adopt predominantly disordered, random-coil-like conformations, whereas the dehydropeptides in gels **6**/**8** and **7**/**9** adopt predominantly extended β-sheet-like conformations. Hydrophobic and π–π stacking interactions between the aromatic moieties of the canonical peptides **10**/**11** give rise to a dense network of thin, flexible fibers with a high number of physical crosslinks. Consequently, hydrogel **10** shows relatively low thermal stability but high mechanical healing. The flexibility of the fibers and the large number of crosslinks facilitate the rapid reformation of the three-dimensional network after shear. In addition to hydrophobic and π–π stacking interactions between aromatic moieties, the β-sheet-like backbone conformation of the dehydropeptides enables cooperative hydrogen bonding within the fibrils. As a result, the dehydropeptide hydrogels form networks with lower entanglement, composed of thicker, bundled and more rigid fibers with a reduced number of crosslinks. Accordingly, dehydropeptide hydrogels **8** and **9** display high mechanical and thermal stability but low mechanical recovery. The reduced number of crosslinks and the limited flexibility of the bundled fibers hinder rapid reorganization of the three-dimensional fibrillar network. Interestingly, the Val-containing dicarboxylic acid hydrogel (**8**) showed a full thermal recovery and higher mechanical healing than the analogous Phe-containing dehydropeptide (**9**). This suggests that the lower steric bulk of the Val side chain may facilitate more efficient supramolecular packing while increasing fiber flexibility [52]. The combined effect of thermal and mechanical stimuli on hydrogel recovery was evaluated using the TMH parameter (*G*′*_r_/G*′*_i_*). Hydrogels **8** and **11** exhibited the best combined recovery, mainly reflecting their high thermal stability. Overall, hydrogel **8** emerges as the most promising candidate for photothermal drug-delivery applications.

#### 2.3.6. Drug-Release Studies

**Figure 14:**
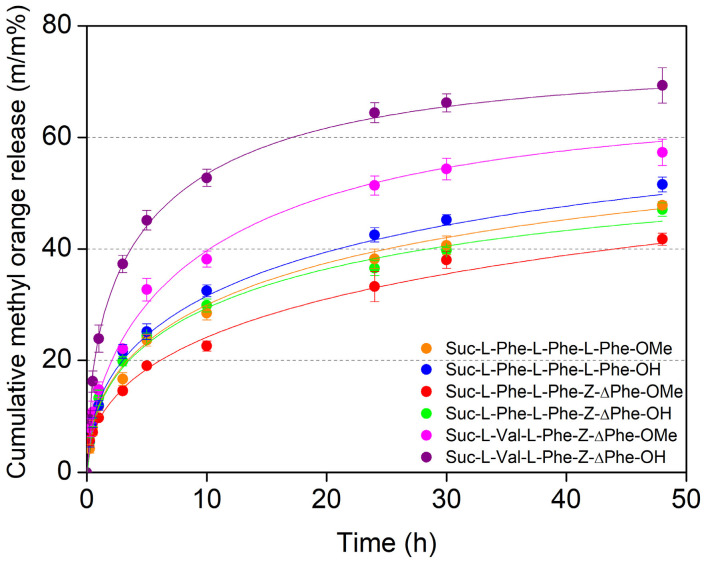
Methyl orange release profiles for (dehydro)peptide hydrogels, fitted to the Weibull model (solid lines).

Methyl orange (MO) was used as a drug model to investigate the release behavior of the peptide hydrogels. MO offers straightforward quantification by absorption spectroscopy in the visible region and high solubility within hydrated self-assembled peptide fibrillar matrices. Its amphiphilic character allows us to infer the effect of hydrophobic and π–π stacking interactions with aromatic nanofibrillar networks on drug release [29,30]. The experimental release profiles were fitted to the Korsmeyer–Peppas (Figure S11 and Table S5) and Weibull mathematical models, which are well-established for describing the drug-release properties of supramolecular hydrogels [53]. The best fitting of the experimental data, the lowest fitting error, was achieved with the Weibull model (Equation (1), Figure 14, Table S5):

(1) $$M\left(t\right)=M_{\infty }\left[1-e^{\left[-\left(kt\right)d\right]}\right]$$

$M_{\infty }$—Represents the total extent of cumulative drug release (value of the asymptotic plateau).

*M*(*t*)*—*Represents the extent of cumulative drug release at any time point.

*k*—Defines the steepness of the curve, measures how fast the plateau is reached.

*d*—Is related to the shape of the curve, which defines the release mechanism: *d* < 0.75 indicates Fickian diffusion; *d*~1 indicates anomalous transport; and *d* > 1 indicates matrix relaxation or gel erosion-controlled release.

The fitted parameters are summarized in Table S5.

The methyl orange (MO) release profiles display typical asymptotic behavior, with the Weibull shape parameter *d* < 0.75, indicating diffusion-controlled release, without significant contributions from matrix relaxation or hydrogel erosion [53]. All hydrogels exhibit biphasic release kinetics. An initial fast release phase is attributed to the Fickian diffusion of loosely bound cargo, at or near the hydrogel surface, followed by a slower sustained release phase, where the diffusion of MO entrapped within the hydrogel interior is slowed by the hydrogel network and by hydrophobic and π–π interactions with the nanofibrillar network. The Suc-L-Val-L-Phe-Z-ΔPhe-OH (**8**) hydrogel exhibits noticeably faster release kinetics and a higher overall extent of cargo release than the other dicarboxylic acid hydrogels **9** and **11,** which is consistent with a lower density network compared to the other hydrogels. The methyl ester hydrogel Suc-L-Val-L-Phe-Z-ΔPhe-OMe (**6**) also shows faster cargo release kinetics and a higher cumulative release extent compared to the hydrogels Suc-L-Phe-L-Phe-Z-ΔPhe-OMe (**7**) and Suc-L-Phe-L-Phe-L-Phe-OMe (**10**), probably due to substantially lower elasticity and lower fibrillar network density. Together, these results highlight the critical role of network density and fibrillar entanglement in governing drug-release behavior.

### 2.4. Proteolytic Stability Assays

**Figure 15:**
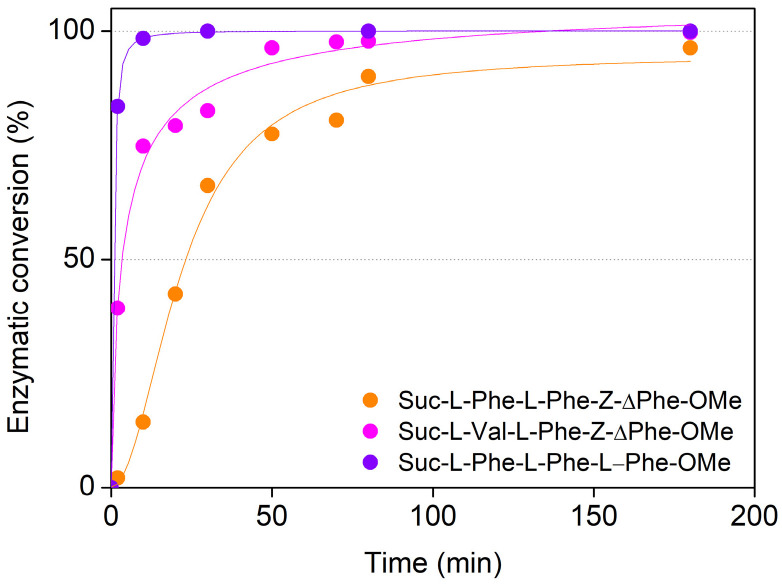
Conversion degree for chymotrypsin-catalyzed hydrolysis of canonical and dehydropeptide methyl esters. The time evolution of the hydrolysis was fitted to a pseudo first-order model (solid lines).

In previous works, we demonstrated that C-deprotected dehydropeptides N-capped with bulky aromatic moieties are resistant to hydrolysis by chymotrypsin [31]. Proteolytic stability was evaluated using pancreatic α-chymotrypsin, a serine endoprotease that preferentially cleaves peptide bonds on the C-terminal side of bulky hydrophobic and aromatic residues (Phe, Tyr, Trp, and to a lesser extent Leu) at the P1 position (Figure 15) [54]. The canonical methyl ester peptide Suc-L-Phe-L-Phe-L-Phe-OMe (**10**) underwent rapid enzymatic hydrolysis of the ester group to yield the corresponding dicarboxylic acid, which underwent further proteolytic cleavage, producing smaller succinylated peptides (Figure S13). The canonical dicarboxylic acid Suc-L-Phe-L-Phe-L-Phe-OH (**11**) followed the same proteolytic degradation pathway (Figure S12 and Table S6). Remarkably, the methyl ester dehydrotripeptides **6** and **7** proved to also be susceptible to chymotrypsin-catalyzed ester hydrolysis, although at a significantly slower rate than the canonical peptide. Significantly, the resulting dicarboxylic acids were revealed to be fully resistant to further proteolysis (Figures S14 and S15).

The experimental conversion values were fitted to a pseudo first-order model (Equation (2)), affording the fitting parameters $C_{\infty }$, the asymptotic conversion extent, and the apparent first-order rate constant for conversion, *k* (Table S7).

(2) $$C\left(t\right)=C_{\infty }\left[1-e^{\left(-kt\right)}\right]$$

The hydrolysis products of the methyl ester dehydropeptides (**6** and **7**) were identified as the corresponding dicarboxylic acids **8** and **9** by a comparison of the HPLC retention times and ESI-MS analysis. No further proteolytic cleavage of the resulting dicarboxylic acid dehydropeptides **8** and **9** was detected by HPLC and ESI-MS over 72 h. Likewise, the dehydro dicarboxylic acids **8** and **9** were found to be completely resistant to proteolysis under the same conditions (Figures S14 and S15). The hydrolysis of the canonical methyl ester is consistent with the well-established esterase activity of chymotrypsin. In contrast, the hydrolysis of the methyl ester dehydropeptides **6** and **7** to dicarboxylic acids is somewhat unexpected. The C-terminal dehydrophenylalanine residue at the P1 position must be accommodated within the enzyme S1 specificity pocket to form a productive enzyme–substrate complex and to enable the stabilization of the tetrahedral intermediate in the oxyanion hole. The observed proteolytic stability of the corresponding dicarboxylic acid dehydropeptides **8** and **9** suggests that the dehydrophenylalanine residue imposes local conformational constraints on the scissile bond that either hinder productive binding within the active site or impair stabilization of the tetrahedral intermediate along the reaction pathway [54]. This side-by-side comparison provides further support to the notion that dehydropeptides exhibit resistance to proteolysis. Protease-catalyzed ester hydrolysis without further proteolytic degradation opens the possibility of protease-triggered hydrogel transformations.

### 2.5. Biocompatibility Studies

**Figure 16:**
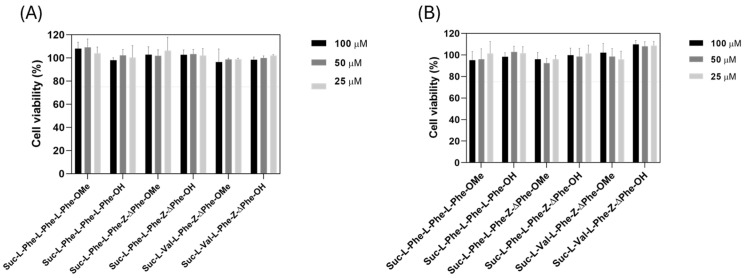
Cell viability of human dermal fibroblast cell line (HDF) (**A**) and PBMCs (**B**) incubated with (dehydro)peptides at different concentrations: 25, 50 and 100 μM.

The biocompatibility profile for the synthesized (dehydro)peptides was evaluated using the human dermal fibroblast cell line (HDF) and peripheral blood mononuclear cells (PBMC) (Figure 16).

All compounds, canonical peptides and dehydropeptides, both as methyl esters and dicarboxylic acids, are essentially devoid of concentration-dependent toxicity towards the adherent fibroblasts cell line and the PBMC immune cells in suspension, up to 100 µM.

**Figure 17:**
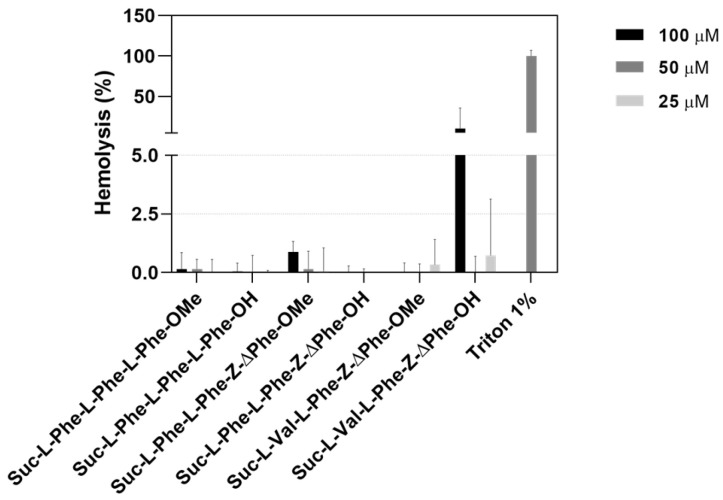
Percentage of hemolysis triggered by (dehydro)peptides at 25, 50 and 100 μM.

As hemocompatibility is a mandatory requisite for biomedical applications of blood-contacting biomaterials, the potential of the (dehydro)peptides to trigger hemolysis was evaluated (Figure 17) [55].

With the exception of the dehydropeptide Suc-L-Val-L-Phe-Z-ΔPhe-OH (**8**), the (dehydro)peptides exhibited negligible hemolytic activity in the concentration range of 25–100 µM, well below the 5% threshold established by the ASTM International protocol E2524-08 for non-hemolytic blood-contacting materials. Dehydropeptide Suc-L-Val-L-Phe-Z-ΔPhe-OH (**8**) showed the highest concentration-dependent hemolytic activity, exceeding the 5% threshold at 100 mM concentration. The negligeable impact of the (dehydro)peptides on cell viability, associated with non-hemolytic properties, warrants further studies towards the development of dehydropeptide-based therapeutic/theragnostic hydrogel platforms.

## 3. Conclusions

In this work, a small library of N-succinylated dehydropeptides and canonical analogs was synthesized, both as methyl esters and as dicarboxylic acids. Combining a robust ensemble of complementary experimental techniques, CD and fluorescence spectroscopy, ThT hydrogel staining, ATR-FTIR, TEM, rheology, and drug-release and proteolysis studies, we demonstrated that dehydropeptide-based hydrogels exhibit enhanced gelation performance and improved mechanical properties, compared to their canonical analogs. Importantly, proteolysis assays with α-chymotrypsin provided unambiguous evidence that dehydroamino acid incorporation endows dehydropeptides with proteolytic resistance. Interestingly, both the canonical and the dehydropeptide esters underwent chymotrypsin-catalyzed ester hydrolysis. However, only their canonical dicarboxylic acids suffered subsequent proteolytic degradation. The dehydropeptide dicarboxylic acids proved to be fully stable against proteolysis. Notably, the FTIR analysis revealed that in the hydrogels’ fibrillar network, the canonical peptides exhibited predominantly disordered random coil-like backbone conformations. In contrast, the dehydropeptides seem to assume mostly extended (antiparallel) β-sheet-like conformations. Interestingly, the dehydropetide dicarboxylic acid hydrogels show a higher content of low-frequency amide I bands than their methyl ester counterparts, which is consistent with a higher content of extended β-sheet-like arrangements, likely promoted by additional hydrogen-bonding and electrostatic interactions involving the carboxylate groups. These distinct self-assembly pathways translate into hydrogel networks with different fiber dimensions, network density, and degree of entanglement, ultimately governing rheological performance, thermal resistance, and diffusion-controlled cargo release. The N-succinylated peptide-based hydrogels span a broad range of elastic moduli that are suitable for diverse biological applications. The negligible cytotoxicity of the peptides towards HaCaT cells supports their potential for biomedical use. The Val dicarboxylic acid hydrogel emerges as a particularly promising candidate for photothermal-triggered drug-delivery applications, due to its excellent thermo-mechanical recovery. Its hemolytic activity deserves further investigation. The N-succinylation strategy provides a versatile platform for the development of functional hydrogels for technological and biomedical applications. Chymotrypsin-catalyzed ester hydrolysis, associated with proteolytic resistance of the corresponding dicarboxylic acids, suggests a potentially useful enzymatic handle for controlling gelation and tuning the hydrogel’s rheological properties.

## 4. Materials and Methods

### 4.1. General Procedures

All chemicals, analytical-grade reagents, and solvents were purchased from Acros Organics (Geel, Belgium) and Sigma-Aldrich (St. Louis, MO, USA) and used as received, unless otherwise stated. When required, solvents were dried using standard procedures. Aqueous solutions were prepared with distilled water. Reaction progress was monitored by thin-layer chromatography (TLC) on Merck silica gel 60 F254 plates (Merck, Darmstadt, Germany), with visualization under UV light and/or iodine staining. Organic layers were dried over anhydrous MgSO_4_.

^1^H and ^13^C NMR spectra were recorded on a Bruker Avance III 400 spectrometer (Bruker BioSpin, Rheinstetten, Germany) (400.13 MHz for ^1^H and 100.62 MHz for ^13^C). DEPT, HSQC, and HMBC experiments were used for signal assignment. Spectra were acquired at 25 °C in DMSO-*d*_6_, and chemical shifts (δ) are reported in ppm, relative to residual solvent signals; coupling constants (*J*) are reported in Hz. Elemental analyses were performed on a LECO analyzer. HRMS (ESI) data were obtained at CACTI (University of Vigo, Vigo, Spain), using a Thermo Finnigan LXQ linear ion trap mass spectrometer (Thermo Fisher Scientific, San Jose, CA, USA).

Full experimental procedures are provided in the Supporting Information.

### 4.2. CAC Determination

Fluorescence measurements were carried out at room temperature, using a Fluorolog-2 spectrofluorometer (Horiba Jobin Yvon, Kyoto, Japan). The critical aggregation concentration (CAC) was determined in phosphate-buffered saline (PBS, pH 7.4), using a range of peptide solutions in the micromolar concentration range. Briefly, peptide stock solutions (330 µM) in PBS (0.1 M, pH 7.4) containing ANS (25 µM) were serially diluted with the same buffer. After equilibration for 30 min at room temperature, fluorescence emission spectra (*λ*_exc_ = 370 nm) were recorded over the 400–600 nm range. The critical aggregation concentration (CAC) was determined by the graphical representation of the maximum ANS fluorescence emission intensity as a function of the logarithm of the peptide concentration. The CAC was obtained from the intersection of the linear regressions corresponding to the monomeric and aggregated states.

### 4.3. Circular Dichroism (CD) Spectroscopy

CD spectra were recorded at 25 °C under a constant nitrogen purge, using a Jasco J-1500 spectropolarimeter (JASCO, Tokyo, Japan). Hydrogelator solutions (0.01 wt%) were prepared as described above and mixed with *D*-glucono-δ-lactone (GDL) at 0.01 wt% for methyl ester hydrogelators or 0.02 wt% for dicarboxylic acid hydrogelators. Samples were loaded into 0.1 mm path-length quartz cuvettes prior to CD measurements. The CD curves were background corrected (PBS) and smoothed using the instrument software.

### 4.4. Molecular Dynamic Simulations

Molecular dynamics (MD) simulations were carried out to better understand the structure of these compounds in aqueous media. The GAFF2 force field, in conjunction with the BCC effective atomic charges [56], were applied to each compound using the AmberTools23 software suite [57], while the TIP3P water model was used for the solvent [58]. All simulations were carried out using the LAMMPS software [59], version dated from the 21st of November of 2023, and geometrical parameters were monitored during the simulations, using the Colvars library [60].

The molecular structure of each compound was obtained from their SMILES representation, using OpenBabel’s 3D structure generation algorithm [61], as implemented in version 3.1.0 [62]. Two states were considered for each compound: a dispersed state (D), in which the solute molecules start the simulation, evenly spread across the simulation box, and an aggregated state (A), where the solute molecules are concentrated in a condensed phase surrounded by water. The initial geometry for each system was generated using the Packmol software, version 21.11.4 [63]. The initial geometry of state D was generated in two successive runs of Packmol: in the first run, 10 solute molecules were dispersed over a 14.5 × 14.5 × 14.5 nm box with periodic boundary conditions and a high tolerance parameter (in order to avoid the random formation of molecular clusters); this initial dispersed geometry was then solvated using 100 k water molecules. For state A, an initial aggregate of 10 solute molecules was placed close to the center of the simulation box and heated to 500 K in the NVT conditions and reflective walls at the six faces of the simulation box for 1 ns, and then allowed to cool to 300 K over 10 ns. The final geometry of this aggregate was then placed at the center of a 14.5 × 14.5 × 14.5 nm box with periodic boundary conditions and solvated using 100 k water molecules. Both the A and D systems of each compound were then allowed to equilibrate at 300 K and 1 atm, using a series of NVT (totaling 3 ns) followed by a series of NPT runs, totaling 5 ns. Convergence of the temperature, density and box dimensions signaled that the equilibration was achieved, and a production run for each system was then performed from 2 ns in NPT conditions at 300 K and 1 atm.

### 4.5. Preparation of Hydrogels

Peptide suspensions (1, 2, 3, or 4 mg) were prepared in ultrapure water (1.0 mL) under magnetic stirring at room temperature. Aqueous NaOH (1.0 M) was added dropwise in small aliquots until complete dissolution was achieved (visual inspection), typically at pH 9–10 (pH paper). The resulting solutions were briefly sonicated (~1 min) and D-glucono-δ-lactone (GDL) was then added. For methyl ester hydrogelators, GDL was added in an amount equal to the peptide mass, whereas for dicarboxylic acid hydrogelators, twice this amount was used. The mixtures were homogenized by gentle inversion and left undisturbed overnight to allow for gelation. Gel formation was evaluated by the vial inversion method. Samples were classified as gels when no flow was observed after 5 min of inversion. The pH of the mature hydrogels was measured using pH paper.

### 4.6. Thioflavin T-Stained Hydrogels and Congo Red Binding Assays

A small amount of mature hydrogel (0.4 wt%) was placed on a glass microscope slide and allowed to air-dry at room temperature overnight. Subsequently, an aqueous Thioflavin T (ThT) solution (20 µL, 50 µM) was added dropwise onto the dried hydrogel and left to dry at room temperature overnight. The resulting stained samples were imaged using a fluorescence microscope using the ThT filter set (excitation/emission in the thioflavin channel). Congo red binding to the peptide hydrogel fibers was assessed by UV–Vis spectroscopy.

Equal volumes (500 µL) of hydrogels and Congo red solution (40 µM in PBS, pH 7.4) were mixed to obtain a final dye concentration of 20 µM. The samples were gently vortexed for 10 s, incubated for 20 min at room temperature, and subsequently centrifuged. The absorption spectra of the supernatants and the control samples (hydrogels without Congo red) were recorded over the 400–700 nm range.

### 4.7. Transmission Electron Microscopy (TEM)

Carbon-coated copper grids (400 mesh) were briefly immersed in the hydrogels. Excess sample was removed by gently blotting with absorbent paper, and the grids were allowed to dry at room temperature. Negative staining was then performed by adding uranyl acetate solution (2% (*v*/*v*), 5 µL) onto the grids. Excess staining was removed by blotting with absorbent paper, and the grids were left to air-dry. Transmission electron microscopy (TEM) images were acquired using a Morgagni 268 transmission electron microscope (FEI, Hillsboro, OR, USA), operated at an accelerating voltage of 200 kV (filament setting: 2).

### 4.8. Fourier Transform Infrared Spectroscopy (FTIR)

ATR-FTIR spectra were acquired on an FT-IR spectrometer (PerkinElmer, Shelton, CT, USA) equipped with a diamond ATR module. Hydrogels for ATR FTIR analysis (0.4 wt%) were prepared in D_2_O as described above. Mature hydrogels were directly placed onto the ATR crystal for measurement. Background spectra were collected from the clean ATR crystal before each sample. Spectra were collected in the transmission mode in the range 4000–400 cm^−1^ at 4 cm^−1^ resolution, averaging 10 scans. The Amide I region (1700–1600 cm^−1^) was selected for deconvolution. Asymmetric least squares (ALS) baseline correction was performed before deconvolution. The number and approximate positions of the overlapping component bands to be used for curve fitting was determined from the second derivative spectra. Nonlinear least-squares curve fitting was performed using OriginPro software 2024b (OriginLab Corporation, Northampton, MA, USA). The Amide I band was deconvoluted into individual components using Gaussian functions. Peak position, full width at half maximum (FWHM), and intensity were iteratively optimized until convergence was achieved and residuals were minimized. The quality of the fit was evaluated by an inspection of the residual distribution and the coefficient of determination (R^2^).

### 4.9. Rheological Studies

A MCR300 stress-controlled rotational rheometer (Anton Paar GmbH, Graz, Austria) was used for the viscoelastic characterization of the hydrogels (0.4 wt%). Rheological experiments were carried out at 25 °C, using the Couette cell geometry (1 mL volume and 0.5 mm gap). Limited sample availability for filling the Couette cell and the long gelation time only allowed for duplicate tests, which show reproducibility well within the 10% rheological accuracy reported for rheometry [64]. Sample homogenization was attained by applying a shear rate of 5 s^−1^ for 1 min to the stress cell loaded with the dehydropeptides’ solutions. Gel formation kinetics was acquired over 10 h by applying a small amplitude oscillatory shear (SAOS), with a frequency of 1 Hz and an amplitude of 0.01%, recording the shear storage (G′) and loss (G″) moduli every 100 s. The elastic modulus of equilibrated gels (*G*′*_i_*) was determined from the frequency sweep measurements (100–0.01 Hz) while applying a constant SAOS amplitude (0.01%). The thermal responsiveness of hydrogels was evaluated by heating–cooling temperature ramps 25–>80 °C (1 °C/min), followed by an equilibration period of 2 h at 25 °C. After thermal recovery, the mechanical stability of the hydrogels was evaluated by strain sweep experiments (0.0001–100%) at 1 Hz. The critical strain (*γ*_*c*_), defined as the strain at which *G*′ = *G*″, corresponding to gel network rupture and fluidization, was determined. Structural recovery following gel break-up was monitored for 1 h as described above for “gel formation kinetics”. The elastic modulus of the healed gel (*G*′*_r_*) was determined from the frequency sweep measurements, as described above. The hydrogels’ thermal healing efficiency was quantified as *H* = *G*′*_h_*/*G*′*_i_*. A thermo-mechanical healing parameter, *TMH* = *G*′*_r_*/*G*′*_i_*, was also calculated to compare the elastic properties of recovered gels with those of freshly equilibrated samples.

### 4.10. Drug-Release Assays

Hydrogels (1.0 mL; 0.4 wt%) loaded with methyl orange (0.2 mM) were prepared as described above. Subsequently, PBS buffer (0,1 M, pH 7.4) (1.0 mL) was carefully layered on top of each hydrogel. At predetermined time points, aliquots (100 µL) of the supernatant were withdrawn and immediately replaced with an equal volume of fresh PBS buffer in order to maintain the sink conditions. Dye concentrations in the collected samples were quantified by UV–Vis spectrophotometry using a Varian Cary 50 Bio spectrophotometer equipped with a microplate reader (Varian, Palo Alto, CA, USA) at 465 nm, based on standard calibration curves. Cumulative release profiles were expressed as the mean percentage release ± SD from three independent experiments. The experimental drug-release profiles were fitted to the Weibull model.

### 4.11. Proteolytic Stability Assays

Proteolytic stability was evaluated using pancreatic α-chymotrypsin, a serine endoprotease that preferentially cleaves peptide bonds on the *C*-terminal side of bulky hydrophobic and aromatic residues (Phe, Tyr, Trp, and to a lesser extent Leu) at the P1 position. Peptide solutions (0.5 mg·mL^−1^ in PBS 1×, pH 7.4) were incubated with α-chymotrypsin (5.1 U·mL^−1^) under magnetic stirring at 25 °C. Reaction aliquots were analyzed by RP-C18 HPLC under isocratic elution (1 mL·min^−1^; MeCN/H_2_O 55:45 containing 0.1% TFA), with UV detection at 260 nm for canonical peptides and 280 nm for dehydropeptides. A JASCO HPLC system (JASCO Inc., Tokyo, Japan) equipped with a PU-480 pump, a UV-4075 UV–Vis detector, and a BS-4001 degasser was used for chromatographic analysis. Data acquisition and processing were carried out using JASCO chromatography software (ChromNAV 2.0). The experimental conversion degree was calculated, monitoring over time the reduction in the peak area of the ester or dicarboxylic acid substrates *C*(*t*)(%) = (1 − *A*_t_/*A*_0_) × 100.

### 4.12. Biocompatibility Studies

Cell lines and PBMC isolation

The human dermal fibroblast (HDF) cell line was kindly provided by the Department of Drug Sciences, University of Pavia. Peripheral blood mononuclear cells (PBMCs) were isolated from peripheral blood samples collected from healthy donors in sterile EDTA-coated tubes. Whole blood was diluted 1:1 with PBS and gently layered onto Ficoll^®^ Paque Plus (Merck, Darmstadt, Germany)) at a 2:1 blood-to-Ficoll ratio. The samples were then centrifuged at 1200× *g* for 20 min at room temperature with the brake off. The PBMC layer at the plasma–Ficoll interface was collected, washed with PBS, treated with ACK lysing buffer (Gibco™) to remove residual erythrocytes, washed again, counted, and used for subsequent experiments. Cells were cultured under standard conditions. HDF cells were maintained in high-glucose Dulbecco’s modified Eagle’s medium (DMEM, Gibco™, Thermo Fisher Scientific, Waltham, MA, USA), whereas PBMCs were cultured in Roswell Park Memorial Institute medium (RPMI-1640, Gibco™). Both media contained heat-inactivated fetal bovine serum (FBS, Sigma-Aldrich, St. Louis, MO, USA) and 1% penicillin-streptomycin (100 U mL^−1^ and 100 µg mL^−1^, respectively). Cells were cultured at 37 °C in a humidified atmosphere containing 5% CO_2_. The culture medium was renewed every 2–3 days, and cells were maintained at 70–80% confluence.

Cell Viability:

HDF and PBMCs were plated in a 96-well plate at a concentration of 1 × 10^4^ or 5 × 10^4^ cells per well in complete DMEM or RPMI, respectively, and were left to adhere for 24 h. Afterwards, the treatments were added and incubated for 48 h at 37 °C, 5% CO_2_. AquaBluer reagent (MultiTarget Pharmaceuticals LLC, Colorado Springs, CO, USA) at a 1:100 dilution in complete DMEM or RPMI medium was added and then incubated for 4 h according to the manufacturer’s instructions. Finally, the 100 μL of the medium with AquaBluer were transferred to a new 96-well plate and the fluorescence intensity (FI) was measured (*λ*_ex_ = 540 nm, *λ*_em_ = 590 nm) using a microplate reader (Biotek, model: SYNERGY H1, Winooski, VT, USA). The percentage of cell viability was calculated by following the formula below:

Cell viability (%) = FI (Cells + Drug) − FI (Drug)/FI (Cells − Culture medium) × 100

Hemolysis:

Whole blood obtained from healthy donors (collected in sterile EDTA-coated tubes, as described above) was used for the hemolysis assay. Briefly, PBS was added in a 3% *w*/*v* ratio and then 80 μL of diluted blood suspension was added in a 96 U-bottom plate (COSTAR^®^, Corning Inc., New York, NY, USA); 80 μL of each drug was added to a final concentration of 25, 50 and 100 μM. The plate was incubated for 4 h at 37 °C, followed by centrifugation at 1000 g for 10 min at 4 °C, and 80 μL of the supernatant were transferred to a new plate of 96 flat bottom wells. Absorbance was read at 558 nm on the Synergy Biotek H1 Microtiter Plate Reader, Winooski, VT, USA. As a positive and negative control, PBS and 1% Triton X-100 (Sigma-Aldrich, San Luis, MO, USA) in PBS were used, respectively. The percentages of hemolysis were calculated using the following formula:

Hemolysis (%) = (Abs Sample × Abs PBS)/(Abs Triton × Abs PBS) × 100

According to the ASTM International protocol E2524-08, 0–2% hemolysis is considered non-hemolytic, 2–5% moderately hemolytic and >5% hemolytic [51].

## Figures and Tables

**Table 1:** ^1^H NMR signals (400 MHz, DMSO-d_6_) attributed to the amide (NH) protons of the (dehydro)peptides.

(Dehydro)peptides	δ (ppm)		
	NH(Phe)_N-ter_	NH(Phe)	NH(Phe)
Suc-L-Phe-L-Phe-L-Phe-OMe (**10**)	8.40	8.00	7.98
Suc-L-Phe-L-Phe-L-Phe-OH (**11**)	8.24	7.99	7.97
	NH(∆Phe)_C-ter_	NH(Phe)_N-ter_	NH(Phe)_int_
Suc-L-Phe-L-Phe-Z-ΔPhe-OMe (**7**)	9.83	8.23	8.01
Suc-L-Phe-L-Phe-Z-ΔPhe-OH (**9**)	9.68	8.49	7.91
Suc-L-Val-L-Phe-Z-ΔPhe-OMe (**6**)	9.79	8.11	7.78
Suc-L-Val-L-Phe-Z-ΔPhe-OH (**8**)	9.61	8.40	8.10

**Table 2:** Calculated CAC values for (dehydro)peptide gelators.

(Dehydro)peptide	CAC (µM)	c*L*ogP *
Suc-L-Phe-L-Phe-L-Phe-OMe (**10**)	155	2.65
Suc-L-Phe-L-Phe-L-Phe-OH (**11**)	144	0.58
Suc-L-Phe-L-Phe-Z-∆Phe-OMe (**7**)	108	3.32
Suc-L-Phe-L-Phe-Z-∆Phe-OH (**9**)	119	2.71
Suc-L-Val-L-Phe-Z-∆Phe-OMe (**6**)	120	2.64
Suc-L-Val-L-Phe-Z-∆Phe-OH (**8**)	100	2.02

* Molinspiration chemoinformatics: https://www.molinspiration.com/ (accessed on 18 December 2025).

**Table 3:** Critical gelation concentration (CGC, wt%) and pH values for mature hydrogels.

Hydrogelator	CGC (wt%)	GDL (wt%)	pH
Suc-L-Phe-L-Phe-L-Phe-OMe (**10**)	0.3	0.3	5.8
Suc-L-Phe-L-Phe-L-Phe-OH (**11**)	0.3	0.6	5.7
Suc-L-Phe-L-Phe-Z-∆Phe-OMe (**7**)	0.2	0.2	5.6
Suc-L-Phe-L-Phe-Z-∆Phe-OH (**9**)	0.2	0.4	5.5
Suc-L-Val-L-Phe-Z-∆Phe-OMe (**6**)	0.2	0.2	5.7
Suc-L-Val-L-Phe-Z-∆Phe-OH (**8**)	0.2	0.4	5.8

**Table 4:** Rheological characterization of the thermo-mechanical stability and self-healing properties of the (dehydro)peptide hydrogels.

(Dehydro)peptide	*G*′*_i_* (kPa)	*G*′*_h_* (kPa)	*γ*_*c*_ (%)	TH (%)	*G*′*_r_* (kPa)	MH (%)	TMH (%)
Suc-L-Phe-L-Phe-L-Phe-OMe (**10**)	256	60	1 ± 0.1	23.4	33	55	12.9
Suc-L-Phe-L-Phe-L-Phe-OH (**11**)	12	18	152 ± 12	150.0	6	34	50.0
Suc-L-Phe-L-Phe-Z-∆Phe-OMe (**7**)	294	45	48 ± 7	15.3	32	71	10.9
Suc-L-Phe-L-Phe-Z-∆Phe-OH (**9**)	259	177	14 ± 2.2	68.3	13	38	5.0
Suc-L-Val-L-Phe-Z-∆Phe-OMe (**6**)	20	16	60 ± 11	80.0	3	18	15.0
Suc-L-Val-L-Phe-Z-∆Phe-OH (**8**)	151	170	7 ± 1.2	112.6	80	47	53.0

## Data Availability

The original contributions presented in this study are included in the article/Supplementary Materials; further inquiries can be directed to the corresponding authors.

## Supplementary Materials

The following supporting information can be downloaded at: https://www.mdpi.com/article/10.3390/gels12040299/s1; Figure S1: Variable-temperature ^1^H-NMR studies (400 MHz; DMSO-*d*_6_) of the amide (NH) proton chemical shifts for (dehydro)peptides: (A) Suc-L-Phe-L-Phe-Z-∆Phe-OMe (**10**); (B) Suc-L-Phe-L-Phe-Z-∆Phe-OH (**11**); (C) Suc-L-Phe-L-Phe-Z-∆Phe-OMe (**7**); (D) Suc-L-Phe-L-Phe-Z-∆Phe-OH (**9**); (E) Suc-L-Val-L-Phe-Z-∆Phe-OMe (**6**); (F) Suc-L-Val-L-Phe-Z-∆Phe-OH (**8**); Figure S2: Experimental methodology for determination of the CAC values for the (dehydro)peptides: (1) Steady-state fluorescence spectra (*λ*_exc_ = 370 nm) in the concentration range 330 to 16 μM in the presence of added ANS (25 μM); (2) semilogarithmic graphical representation of the concentration dependence of the fluorescence maximum emission intensity of the ANS probe (*λ*_ex_ = 370 nm). (A) Suc-L-Phe-L-Phe-L-Phe-OMe (**10**); (B) Suc-L-Phe-L-Phe-L-Phe-OH (**11**); C) Suc-L-Phe-L-Phe-Z-∆Phe-OH (**9**); (D) Suc-L-Val-L-Phe-Z-∆Phe-OMe (**6**); (E) Suc-L-Val-L-Phe-Z-∆Phe-OH (**8**); Figure S3: UV-VIS spectra of the (dehydro)peptides (300 μM, PBS pH7.4); Figure S4: High Tension (HT) spectra corresponding to the CD spectra shown in Figure 4 in the manuscript; Figure S5: Box plot showing the distribution of the distance between the molecular centres for the (dehydro)peptides in the Dispersed (D) and Aggregated (Cluster) (A) states: **10**—Suc-L-Phe-L-Phe-L-Phe-OMe; **11**—Suc-L-Phe-L-Phe-L-Phe-OH; **7**—Suc-L-Phe-L-Phe-Z-∆Phe-OMe; **9**—Suc-L-Phe-L-Phe-Z-∆Phe-OH; **6**—Suc-L-Val-L-Phe-Z-∆Phe-OMe; **8**—Suc-L-Val-L-Phe-Z-∆Phe-OH.; Figure S6: Distribution of peptide-peptide (A) and peptide-water (B) hydrogen bond counting in the production MD simulations for the studied dehydropeptides in the Dispersed and Aggregate state: **10**—Suc-L-Phe-L-Phe-L-Phe-OMe; **11**—Suc-L-Phe-L-Phe-L-Phe-OH; **7**—Suc-L-Phe-L-Phe-Z-∆Phe-OMe; **9**—Suc-L-Phe-L-Phe-Z-∆Phe-OH; **6**—Suc-L-Val-L-Phe-Z-∆Phe-OMe; **8**—Suc-L-Val-L-Phe-Z-∆Phe-OH; Figure S7: Depiction of molecular pairs of dehydropeptide Suc-L-Val-L-Phe-Z-∆Phe-OH (**8**) in the aggregate phase during the MD simulations, exemplifying: (a) the approximate geometry of the T-shaped π–π interaction between the aromatic rings of Phe; (b) intermolecular hydrogen bond between the NH of Val and the C=O of the Val residue of another molecule; Figure S8: UV-Vis absorption spectra of Congo red in PBS in the absence (black trace) and presence (colored traces) of peptide hydrogel fibers; Figure S9: The deconvolution of the Amide I region (1700–1600 cm^−1^) of the FTIR spectra was accomplished using OriginPro software. Baseline correction was performed using the ALS protocol. The initial peak positions were determined from the second-derivative of the spectrum. The experimental curve (black line) was fitted by nonlinear least-squares analysis using Gaussian functions to resolve overlapping component bands (green curves). The red curve represents the cumulative fit (sum of all Gaussian components), demonstrating good agreement with the experimental data. Peak positions, full width at half maximum (FWHM), and intensities were iteratively optimized until convergence was achieved minimizing the residual error. The resolved sub-bands are assigned to different protein secondary structure contributions within the Amide I envelope. (A) Suc-L-Phe-L-Phe-L-Phe-OMe (**10**); (B) Suc-L-Phe-L-Phe-L-Phe-OH (**11**); (C) Suc-L-Phe-L-Phe-Z-∆Phe-OMe (**7**); (D) Suc-L-Phe-L-Phe-Z-∆Phe-OH (**9**); (E) Suc-L-Val-L-Phe-Z-∆Phe-OMe (**6**); (F) Suc-L-Val-L-Phe-Z-∆Phe-OH (**8**); Figure S10: Detailed experimental protocol implemented for the characterization of (dehydro)tripeptide hydrogels: (A) gelation kinetics; (B) Mechanical spectrum, frequency sweep, for equilibrated gels; (C) Temperature cycling; (D) Strain sweep on *rested gels* after temperature cycling; (E) Hydrogel reformation kinetics after mechanical breakdown; (F) Mechanical spectrum, frequency sweep, for reformed hydrogels. (6.1) Suc-L-Phe-L-Phe-L-Phe-OMe (**10**); (6.2) Suc-L-Phe-L-Phe-L-Phe-OH (**11**); (6.3) Suc-L-Phe-L-Phe-Z-∆Phe-OH (**9**); (6.4) Suc-L-Val-L-Phe-Z-∆Phe-OMe (**6**); (6.5) Suc-L-Val-L-Phe-Z-∆Phe-OH (**8**); Figure S11: Methyl Orange-release profiles for (dehydro)peptide hydrogels fitted to the Korsmeyer-Peppas model (solid lines). Figure S12: Conversation degree for chymotrypsin-catalyzed hydrolysis of the canonical dicarboxylic acid Suc-L-Phe-L-Phe-L-Phe-OH (**11**). The time evolution of the hydrolysis was fitted to a pseudo first order model (solid line); Figure S13: RP HPLC profiles for chymotrypsin-catalysed hydrolysis: (A) Suc-L-Phe-L-Phe-L-Phe-OMe (**10**); (B) Suc-L-Phe-L-Phe-L-Phe-OH (**11**). (C) MS spectrum of the hydrolysis product of ester Suc-L-Phe-L-Phe-L-Phe-OMe (**11**); Figure S14: HPLC profiles for chymotrypsin-catalysed hydrolysis: (A) Suc-L-Phe-L-Phe-Z-∆Phe-OMe (**7**) and (B) Suc-L-Phe-L-Phe-Z-∆Phe-OH (**9**). (C) MS spectrum of the hydrolysis product of ester Suc-L-Phe-L-Phe-Z-∆Phe-OMe; Figure S15: HPLC profiles for chymotrypsin-catalysed hydrolysis: (A) Suc-L-Val-L-Phe-Z-∆Phe-OMe (**6**) and (B) Suc-L-Val-L-Phe-Z-∆Phe-OH (**8**). (C) MS spectrum of the hydrolysis product of ester Suc-L-Val-L-Phe-Z-∆Phe-OMe (**6**); Table S1: Chemical shift temperature gradient values (∆δ/∆T, ppb/K) for the (dehydro)peptide amides (400 MHz, DMSO-*d*_6_); Table S2: (Dehydro)peptides’ solubility; Table S3: Solvent Accessible Surface Area (SASA, given as average ± standard deviation) for the dehydropeptides in the Dispersed and Aggregate states; Table S4: Deconvolution of the amide I region of the ATR FTIR spectra; Table S5: Best parameters for the fitting of the experimental Methyl Orange-release profiles of the (dehydro)peptide hydrogels to the *Weibull* and *Korsmeyer-Peppas* models; Table S6: Best-fit parameters for the chymotrypsin-catalyzed hydrolysis of canonical dicarboxylic acid Suc-L-Phe-L-Phe-L-Phe-OH (**11**) to a pseudo first order model (Equation (2) in the main text); Table S7. Best-fit parameters for the fitting of the experimental conversation rate for the chymotrypsin-catalysed methyl ester hydrolysis to a pseudo first order model (Equation (2) in the main text).
