# Supplementary material for: N-Succinylated Canonical vs. Dehydropeptides: Contrasting Self-Assembly Pathways and Hydrogel Properties
Source: Gels. 2026 Apr 1;12(4):299. doi: 10.3390/gels12040299 (PMC13115676; doi:10.3390/gels12040299)
Supplement: Supplementary file 1 [file gels-12-00299-s001.zip › gels-4196284-supplementary.pdf]

Supplementary Information

N-Succinylated Canonical vs. Dehydropeptides: Contrasting Assembly Pathways and Hydrogel Properties

Teresa Pereira[1], André F. Carvalho[1], Andreia Patrícia Magalhães[4], Filipe Teixeira[1], David M. Pereira[2], Loic Hilliou[3], Antero Abrunhosa[5], Manuel Bañobre-López[4], Paula M. T. Ferreira [1,*] and José A. Martins [1,*]

[1] Center of Chemistry, University of Minho, 4710-057 Braga, Portugal
[2] REQUIMTE/LAQV, Laboratório de Farmacognosia, Departamento de Química, Faculdade de Farmácia, Universidade do Porto, R. Jorge Viterbo Ferreira, n 228, 4050-313 Porto, Portugal
[3] Institute for Polymers and Composites, University of Minho, 4800-058 Guimarães, Portugal
[4] Advanced (Magnetic) Theranostic Nanostructures Lab, International Iberian Nanotechnology Laboratory (INL), Av. Mestre José Veiga s/n, 4715-330 Braga, Portugal. manuel.banobre@inl.int (M.B.); Andreia Magalhães (A.M.)
[5] Coimbra Institute for Biomedical Engineering and Translational Research (CIBIT) / Institute for Nuclear Sciences Applied to Health (ICNAS), University of Coimbra, Coimbra 3000-548, Portugal
* pmf@quimica.uminho.pt (PMF); jmartins@quimica.uminho.pt (JAM)

1. *Chemical Synthesis*

1.1. Synthesis of Boc-L-Phe-(D,L)-β-hydroxyphenylalanine-OMe (2).

In a general procedure, Boc-Phenylalanine-OH (1.24 g, 4.67 mmol) was dissolved in MeCN (50 mL) in an ice bath under magnetic stirring. HBTU (4.77 mmol, 1.81 g) was added in small portions over 5 minutes. The reaction mixture was left stirring for 15 minutes at the ice temperature, before adding (*D,L*)-phenilserine methyl ester **1** (as hydrochloride form) (1.08 g, 4.66 mmol) and TEA (13.95 mmol, 1.94 mL). The reaction mixture was allowed to reach room overnight, filtered and the solvent was evaporated under reduced pressure. The residue was dissolved in ethyl acetate (100 mL) and filtered. The filtrated was washed with KHSO4 (3 x 50 mL), NaHCO3 (3 x 50 mL) and brine (50 mL). The organic phase was dried (MgSO4), filtrated and the solvent was evaporated under reduced pressure. Dipeptide Boc-L-Phe-(D,L)-β-hydroxyphenylalanine-OMe (**2**) was obtained as a white foam (1.98 g, 4.47 mmol, 96 %).

^{1}H-NMR (400 MHz, DMSO): δ=1.27 (s,9H, Boc), 2.37-2.79 (m, 4H, CH2β Phe), 3.66 (s, 3H, OMe), 4.58 (m, 1H, CHα Phe), 5.17 (t, J = 3.8 Hz, 1H, CHα Phe), 5.97 (dd, J = 4.8 Hz and J = 8.8 Hz, 1H, OH), 6.70 (d, J = 8.8 Hz, 1H, CHβ PheSer), 6.99-7.29 (m, 10H, CH ar), 8.01 (d, J = 8.4 Hz, 1H, NH Phe), 8.22 (d, J = 8.8 Hz, 1H, NH PheSer).

1.2. Synthesis of Boc-protected dehydrodipeptide Boc-L-Phe-Z-ΔPhe-OMe (3)

In a general procedure, herein illustrated for dehydrodipeptide Boc-L-Phe-Z-ΔPhe-OMe (**3**), dipeptide (**2**) (1.97 g, 4.45 mmol) was dissolved in dry MeCN (10 mL) under stirring at room temperature before adding in small portions Boc_2O (1.21 g, 5.54 mmol) and DMAP (0.07 g; 0.57 mmol, 10% mol). The formation of the carbonate ester was assessed by ^{1}H NMR by monitoring

the up field shift of the signal attributed to the α-CH proton of the β-hydrophenylalanine block and the disappearance of the β-OH signal at δ x ppm. Upon completion, *N,N,N',N'*-tetramethylguadinine (2 % in volume) was added, and the reaction was kept under magnetic stirring at room temperature overnight. Completion of the dehydration step was checked by ^{1}H NMR by confirming the disappearance of the signal attributed to the α-CH proton of the β-hydrophenylalanine block. The solvent was evaporated under reduced pressure, the residue was dissolved in ethyl acetate (100 mL) and was washed with $KHSO_4$ (3 x 50 mL), $NaHCO_3$ (3 x 50 mL) and brine (50 mL). The organic phase was dried ($MgSO_4$) filtrated and the solvent was evaporated under reduced pressure to obtain dehydrodipeptide **Boc-L-Phe-Z-ΔPhe-OMe** (**3**) as a white foam (1.56 g, 3.68 mmol, 83 %).

^{1}H-NMR (400 MHz, DMSO): δ=1.31 (s, 9H, Boc), 2.79 (dd, J = 10.6 Hz and J = 13.6 Hz, 1H, CHβ Phe), 3.04 (dd, *J* = 3.6 Hz and *J* = 13.6 Hz, 1H, CHβ Phe), 3.69 (s, 3H, OMe), 4.33 (ddd, *J* = 4 Hz, 10.8 Hz and 14.4 Hz, 1H, CHα Phe), 7.07 (d, *J* = 8.4 Hz, 1H, CHβ PheSer), 7.19-7.37 (m, 10H, CH ar), 7.67 (m, 1H, NH Phe), 9.79 (s, 1H, NH PheSer).

1.3. Synthesis of Boc-protected canonical tripeptide Boc-L-Phe-L-Phe-L-Phe-OMe (10) and dehydro-tripeptides Boc-L-Xaa-L-Phe-Z-ΔPhe-OMe (4- Xaa= Val and 5- Xaa = Phe).

In a general procedure, herein illustrated for dehydrotripeptide Boc-L-Phe-L-Phe-Z-ΔPhe-OMe (**5**), dehydropeptide **Boc-L-Phe-Z-ΔPhe-OMe** (**3**) (1.30g, 3.06 mmol) was dissolved in TFA (1 mL/mmol) and the solution was kept undisturbed for 1 hour. DCM (~10 mL) was added and the solvent was evaporated under reduced pressure to afford a thick oil. The oil was co-evaporated with diethyl ether (3x10 ml) and dissolved in MeCN (~ 40 ml). Boc-*L*-Phe (0.82 g, 3.10 mmol) was dissolved in MeCN (40 mL) in an ice bath under stirring, followed by addition of HBTU (3.41 mmol, 1.29 g) in small portions over 5 minutes. The reaction mixture was kept under stirring for 15 minutes. The TFA-deprotected dehydrodipeptide solution in MeCN was added to the phenylalanine active ester solution followed by dropwise addition of TEA (9.20 mmol, 1.30 mL) until pH 7-8 (pH paper). The reaction mixture was allowed to reach room temperature and was kept under magnetic stirring overnight. The solvent was evaporated under reduced pressure, the residue was dissolved in ethyl acetate (100 mL) and was washed with $KHSO_4$ (3 x 50 mL), $NaHCO_3$ (3 x 50 mL) and brine (50 mL). The organic phase was dried ($MgSO_4$) filtrated and the solvent was evaporated under reduced pressure to afford Boc protected **Boc-L-Phe-L-Phe-Z-ΔPhe-OMe** (**5**) as a white foam (1.47 g, 2.57 mmol, 84 %).

^{1}H-NMR (400 MHz, DMSO): δ= 1.25 (s, 9H, Boc), 2.60 - 3.15 (m, 4H, CHβ Phe), 3.69 (s, 3H, OMe), 4.16 (m, 1H, CHα Phe), 4.73 (m, 1H, CHα Phe), 7.16 – 7.37 (m, 15H, CH ar), 8.16 (d, *J* = 8 Hz, 1H, NH), 9.89 (s, 1H, NH PheSer).

^{13}C-NMR (100 MHz, DMSO): δ= 35.16 (CH_2β Phe), 36.64 (CH_2β Phe), 51.54 (OMe), 53.34 (CHα Phe), 53.80 (CHα Phe), 124.63 – 131.26 (C ar), 163.83 (C OMe), 170.62 (C NH), 171.89 (C NH), 172.74 (C ΔNH).

Boc-L-Val-L-Phe-Z-ΔPhe-OMe (4) was obtained as a white foam (1.15 g, 2.20 mmol, 85 %), following the procedure detailed above using Boc-L-Phe-Z-ΔPhe-OMe (**3**) (1.10 g, 2.59 mmol) and Boc-*L*-Val (0.56 g, 2.62 mmol).

^{1}H-NMR (400 MHz, DMSO): δ= 0.70 (m, 6H, CH_3 Val), 1.35 (s, 9H, Boc), 1.98 (s, 1H, CHβ Val), 2.83 (m, 1H, CHβ Phe), 3.10 (m, 1H, CHβ Phe), 3.77 (s, 3H, OMe), 4.01 (m, 1H, CHα Phe), 4.77 (m, 1H, CHα Phe), 6.60 (d, *J* = 9.2 Hz, 1H, CHβ DPhe), 7.19 – 7.58 (m, 10H, CH ar), 8.05 (d, *J* = 7.6 Hz, 1H, NH), 8.15 (d, *J* = 7.6 Hz, 1H, NH), 9.89 (s, 1H, ΔNH).

^{13}C-NMR (100 MHz, DMSO): δ= 16.57 (CH_3 Val), 18.33 (CH Val), 19.37 (CH_3 Val), 36.11 (CH_2β Phe), 50.92 (C OMe), 52.63 (CHα Phe), 56.78 (CHα Val), 125.25 – 136.76 (CH ar and CHβ DPhe), 166.35 (C OMe), 170.54 (C NH), 170.89 (C NH), 172.32 (C ΔNH).

Boc-L-Phe-L-Phe-L-Phe-OMe (**10**) was obtained as a white foam (1.83 g, 3.19 mmol, 87 %) following the procedure detailed above using Boc-L-Phe-L-Phe-OMe (**2**) (1.10 g, 2.59 mmol) and Boc-*L*-Phe (0.970 g, 3.66 mmol).

^{1}H-NMR (400 MHz, DMSO): δ=1.26 (s,9H, Boc), 2.49-3.02 (m, 6H, CH2β Phe), 3.57 (s, 3H, OMe), 4.08 (m, 1H, CHα Phe), 4.52 (m, 1H, CHα Phe), 4.60 (m, 1H, CHα Phe), 6.83 (d, J = 8.8 Hz, 1H, NH), 7.14-7.28 (m, 10H, CH ar), 7.91 (d, J = 6.8 Hz, 1H, NH), 8.53 (d, J = 7.2 Hz, 1H, NH).

1.4. Synthesis of N-succinylated canonical tripeptide methyl ester Suc-L-Phe-L-Phe-L-Phe-OMe (11) and dehydro-tripeptides Suc-L-Xaa-L-Phe-Z-ΔPhe-OMe (6- Xaa= Val and 7- Xaa= Phe).

In a general procedure, herein illustrated for dehydrotripeptide **Suc-L-Phe-L-Phe-Z-ΔPhe-OMe** (**7**), dehydropeptide **Boc-L-Phe-L-Phe-Z-ΔPhe-OMe (7)** (1.20 g, 2.10 mmol) was dissolved in TFA (1 mL/mmol) and the solution was kept undisturbed for 1 hour. DCM (~10 mL) was added and the solvent was evaporated under reduced pressure. This procedure was repeated several times to obtain tick oil. The oil was dissolved in dry pyridine (~5 mL) with stirring under inert atmosphere (N_2) before adding succinic anhydrous (0.63 g, 6.30 mmol). The reaction mixture was kept overnight under magnetic stirring at room temperature. Diethyl ether (~ 100 ml) was added under magnetic stirring to the reaction mixture in small volumes resulting in the appearance of a copious white precipitate. The precipitate was filtered, washed with ether and stirred with aqueous HCl 1 M (~ 50 ml) for 2 hours. The precipitate was recovered by filtration, washed with water until neutrality, and dried under vacuum to afford succinylated dehydrotripeptide **Suc-L-Phe-L-Phe-Z-ΔPhe-OMe** (**7**) (0.95 g, 1.66 mmol, 79 %) as a white solid. HRMS (ESI) m/z: $[M + H]^+$ for C32H33N3O7: cal 572.2391; found 572.2393.

^{1}H NMR (400 MHz, DMSO): δ= 2.26 (m, 4H, CH suc), 2.67-3.15 (m, 4H, CHβ Phe), 3.70 (s, 3H, OMe), 4.48-4.52 (m, 1H, CHα Phe), 4.67-4.70 (m, 1H, CHα Phe), 7.18-7.62 (m, 15H, CH aromatics), 8.01 (d, J = 8.8 Hz, 1H, NH), 8.24 (d, J = 8 Hz, 1H, NH), 9.83 (s, 1H, NH ΔPhe).

^{13}C-NMR (100 MHz, DMSO): δ= 28.94 (CH_2 succ), 29.74 (CH_2 succ), 36.96 ($CH_2\beta$ Phe), 37.46 ($CH_2\beta$ Phe), 52.16 (OMe), 53.60 (CHα Phe), 54.09 (CHα Phe), 125.93 – 133.18 (C ar), 165.31 (C OMe), 171.42 (C NH), 172.19 (C NH), 173.76 (C ΔNH).

Suc-L-Val-L-Phe-Z-ΔPhe-OMe (**6**) was obtained as a white solid (0.75 g, 1.43 mmol, 82 %) following the procedure detailed above using **Boc-L-Val-L-Phe-Z-ΔPhe-OMe** (**4**) (0.90 g, 1.72 mmol) and succinic anhydrous (0.59 g, 5.0 mmol). HRMS (ESI) m/z: $[M + H]^+$ for C28H33N3O7: cal 524.2391; found 524.2389.

^{1}H NMR (400 MHz, DMSO): δ= 2.26 (m, 4H, CH suc), 2.67-3.15 (m, 4H, CHβ Phe), 3.70 (s, 3H, OMe), 4.48-4.52 (m, 1H, CHα Phe), 4.67-4.70 (m, 1H, CHα Phe), 7.18-7.62 (m, 15H, CH aromatics), 8.01 (d, J = 8.8 Hz, 1H, NH), 8.24 (d, J = 8 Hz, 1H, NH), 9.83 (s, 1H, NH ΔPhe).

^{13}C-NMR (100 MHz, DMSO): δ= 28.94 (CH_2 succ), 29.74 (CH_2 succ), 36.96 ($CH_2\beta$ Phe), 37.46 ($CH_2\beta$ Phe), 52.16 (OMe), 53.60 (CHα Phe), 54.09 (CHα Phe), 125.93 – 133.18 (C ar), 165.31 (C OMe), 171.42 (C NH), 172.19 (C NH), 173.76 (C ΔNH).

Suc-L-Phe-L-Phe-L-Phe-OMe (**11**) was obtained as a white solid (1.55 g, 2.70 mmol, 89 %) following the procedure detailed above using **Boc-L-Phe-L-Phe-L-Phe-OMe** (**10**) (1.56 g, 3.66 mmol) and succinic anhydride (0.957 g, 9.57 mmol). HRMS (ESI) m/z: $[M + H]^+$ for C32H35N3O7: cal 574.2912; found 574.2911.

^{1}H-NMR (400 MHz, DMSO): δ= 2.49 (m, 1H, CH2β Phe), 2.57-3.03 (m, 8H, succ and CH2β Phe), 3.36 (m, 1H, CH2β Phe), 3.57 (s, 3H, OMe), 4.10 (m, 1H, CHα Phe), 4.50 (m, 1H, CHα Phe), 4.60 (m, 1H, CHα Phe), 6.83 (d, J = 8.8 Hz, 1H, NH), 7.14-7.28 (m, 10H, CH ar), 7.91 (d, J = 8.4 Hz, 1H, NH Phe), 8.53 (d, J = 7.6 Hz, 1H, NH Phe).

^{13}C-NMR (100 MHz, DMSO): δ= 29.09 (CH_2 succ), 29.92 (CH_2 succ), 36.65 ($CH_2\beta$ Phe), 37.33 ($CH_2\beta$ Phe), 37.47 ($CH_2\beta$ Phe), 51.81 (OMe), 53.55 (CHα Phe), 53.61 (CHα Phe), 53.72 (CHα Phe), 126.11 – 129.20 (C ar), 136.96 (C NH), 137.52 (C NH), 137.88 (C NH), 171.61 (C=O OMe), 173.76 (C=O OH).

1.5. Synthesis of canonical N-succinylated tripeptide dicarboxylic acid Suc-L-Phe-L-Phe-L-Phe-OH (12) and dicarboxylic acid dehydro-tripeptides Suc-L-Xaa-L-Phe-Z-ΔPhe-OH (8- Xaa= Val and 9- Xaa= Phe).

In a general procedure, herein illustrated for dehydrotripeptide **Suc-L-Phe-L-Phe-Z-ΔPhe-OH** (**9**), dehydropeptide **Suc-L-Phe-L-Phe-Z-ΔPhe-OMe (7)** (0.5 g, 0.87 mmol) was dissolved in dioxane (5 mL) and NaOH (1 M, 1 mL) (pH~11). The reaction mixture was kept under magnetic stirring at room temperature. The reaction was monitored by RP HPLC (Silica C18, isocratic elution MeCN/H_2O, 50:50, 0,1% TFA, 1ml/min, detection at 280 nm). Upon completion, the reaction mixture was diluted with water (~ 10 ml) and the dioxane solvent was evaporated under reduced pressure. The aqueous phase (~15 ml) was adjusted to pH around 3-4 (pH paper)

with aqueous HCl (3 M) resulting in the formation of a white precipitate. The precipitate was filtered, washed with water to neutrality (pH paper) and dried under vacuum to afford a white solid (0.42 g, 0.75 mmol, 86 %). HRMS (ESI) m/z: $[M + H]^+$ for C31H31N3O7: cal 558.2235; found 558.2235.

^{1}H NMR (400 MHz, DMSO): δ= 2.21 (m, 4H, CH suc), 2.79-2.98 (m, 4H, CHβ), 4.5 (m, 1H, CH*α*), 4.66 (m, 1H, CH*α*), 6.95 (m, 1H, CHβDPhe), 7.12-7.62 (m, 15H, CH aromatics), 7.93 (d, *J* = 8.4 Hz, 1H, NH), 8.51 (d, *J* = 8.8 Hz, 1H, NH), 9.65 (s, 1H, NH ΔPhe).

^{13}C-NMR (100 MHz, DMSO): δ= 29.03 (CH_2 succ), 29.88 (CH_2 succ), 37.22 (CH_2β Phe), 37.80 (CH_2β Phe), 53.76 (CH*α* Phe), 54.19 (CH*α* Phe), 126.05 – 137.93 (C ar), 166.14 (C OH), 170.76 (C NH), 171.08 (C NH), 173.72 (C ΔNH).

Suc-L-Val-L-Phe-Z-ΔPhe-OH (**8**) was obtained as a white solid (0.35 g, 0.68 mmol, 89 %) following the procedure described above using **Suc-L-Val-L-Phe-Z-ΔPhe-OMe** (**7**) (0.40 g, 0.76 mmol) and NaOH (1 M) (1 mL). HRMS (ESI) m/z: $[M + H]^+$ for C27H31N3O7: cal: 510.2237; found 510.2233.

^{1}H NMR (400 MHz, DMSO: δ= 0.73 (d, *J* = 7.2 Hz, 6H, CH_3 Val), 1.90 (s, 1H, CHβ Val), 2.38 (m, 4H, CH_2 succ), 2.83 (m, 1H CH_2β Phe), 3.14 (m, 1H CH_2β Phe), 4.14 (m, 1H, CH*α* Val), 4.67 (m, 1H, CH*α* Phe), 7.18 – 7.62 (m, 11H, CH ar and CHβ DPhe), 7.79 (d, *J* = 8.8 Hz, 1H, NH), 8.11 (d, *J* = 8.4 Hz, 1H, NH), 9.61 (s, 1H, ΔNH).

^{13}C-NMR (100 MHz, DMSO): δ= 17.92 (CH_3 Val), 18.89 (CH Val), 19.17 (CH_3 Val), 28.91 (CH_2 succ), 29.27 (CH_2 succ), 36.89 (CH_2β Phe), 54.15 (CH*α* Phe), 57.58 (CH*α* Val), 126.22 – 137.99 (CH ar and CHβ DPhe), 166.14 (C OH), 171.13 (C NH), 171.30 (C NH), 173.87 (C ΔNH).

Suc-L-Phe-L-Phe-L-Phe-OH (**12**) was obtained as a white solid (0.47 g, 0.84 mmol, 92%). following the procedure described above using **Suc-L-Phe-L-Phe-L-Phe-OMe** (**11**) (0.52 g, 0.91 mmol) and NaOH (1 M) (1 mL). HRMS (ESI) m/z: $[M + H]^+$ for C31H33N3O7: cal 560.2391; found 560.2390.

^{1}H-NMR (400 MHz, DMSO): δ= 2.49-3.02 (m, 6H, CH2β Phe), 3.57 (s, 3H, OMe), 4.10 (m, 1H, CH*α* Phe), 4.50 (m, 1H, CH*α* Phe), 4.60 (m, 1H, CH*α* Phe), 6.83 (d, J = 8.8 Hz, 1H, NH), 7.14-7.28 (m, 10H, CH ar), 7.91 (d, J = 8.4 Hz, 1H, NH Phe), 8.53 (d, J = 7.6 Hz, 1H, NH Phe).

^{13}C-NMR (100 MHz, DMSO): δ= 29.12 (CH_2 succ), 29.95 (CH_2 succ), 36.66 (CH_2β Phe), 37.33 (CH_2β Phe), 37.48 (CH_2β Phe), 53.51 (CH*α* Phe), 53.64 (CH*α* Phe), 53.73 (CH*α* Phe), 126.13 – 129.25 (C ar), 136.98 (C NH), 137.54 (C NH), 137.90 (C NH), 171.63 (C=O OH), 173.78 (C=O OH).

2. Variable-temperature ^{1}H-NMR studies

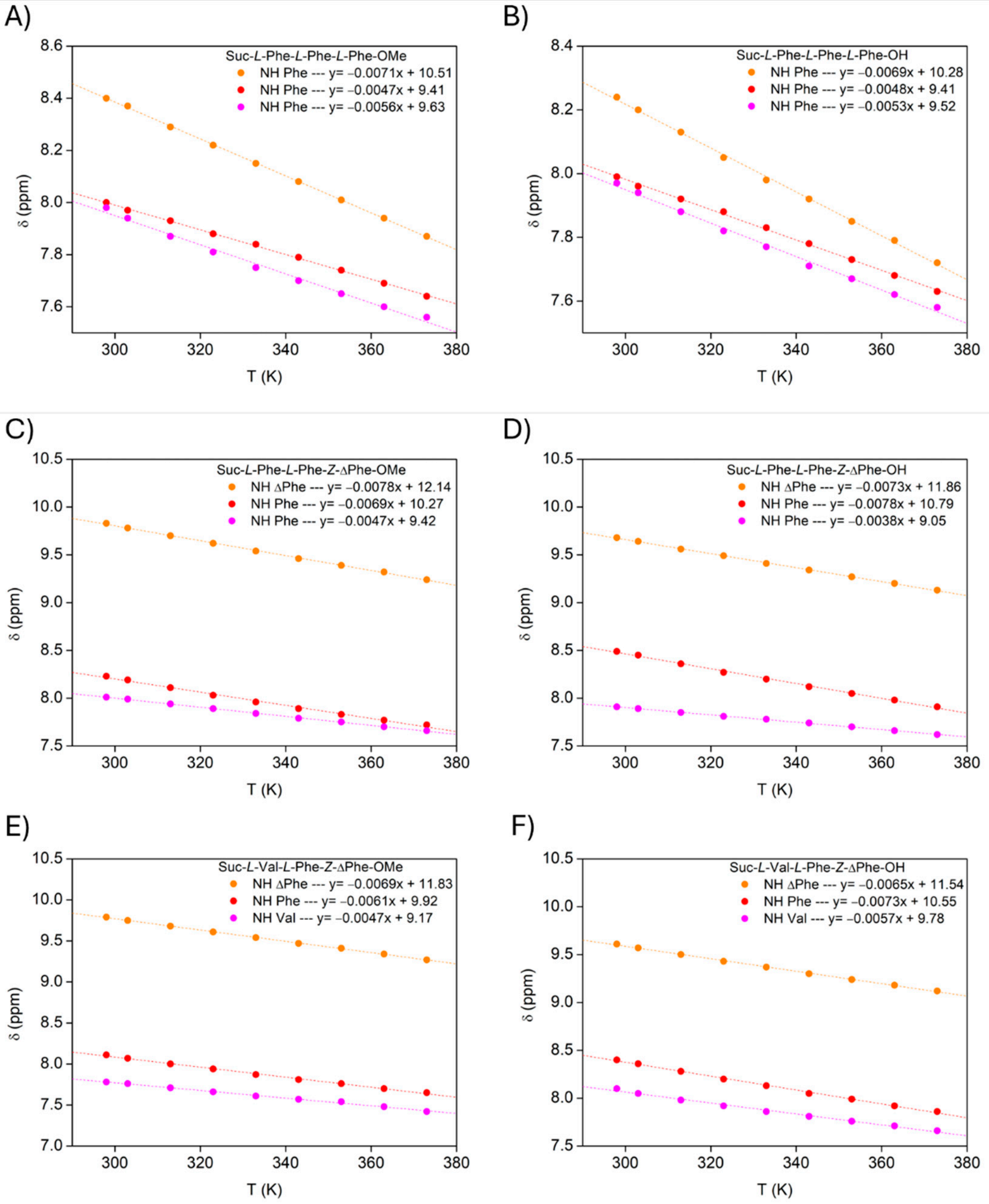

Figure S1. Variable-temperature ^{1}H-NMR studies (400 MHz; DMSO-d_6) of the amide (NH) proton chemical shifts for (dehydro)peptides: A) Suc-L-Phe-L-Phe-Z-ΔPhe-OMe (**10**); B) Suc-L-Phe-L-Phe-Z-ΔPhe-OH (**11**); C) Suc-L-Phe-L-Phe-Z-ΔPhe-OMe (**7**) ; D) Suc-L-Phe-L-Phe-Z-ΔPhe-OH (**9**); E) Suc-L-Val-L-Phe-Z-ΔPhe-OMe (**6**); F) Suc-L-Val-L-Phe-Z-ΔPhe-OH (**8**).

Table S1. Chemical shift temperature gradient values (Δδ/ΔT, ppb/K) for the (dehydro)peptide amides (400 MHz, DMSO-d_6)

(Dehydro)peptides	Δδ/ΔT (ppb)		
	NH Phe	NH Phe	NH Phe
Suc-L-Phe-L-Phe-L-Phe-OMe	-7.1	-4.7	-5.6
Suc-L-Phe-L-Phe-L-Phe-OH	-6.9	-4.8	-5.3
	NH ΔPhe	NH Phe/Val	NH Phe
Suc-L-Phe-L-Phe-Z-ΔPhe-OMe	-7.8	-6.9	-4.7
Suc-L-Phe-L-Phe-Z-ΔPhe-OH	-7.3	-7.8	-3.8
Suc-L-Val-L-Phe-Z-ΔPhe-OMe	-6.9	-6.1	-4.7
Suc-L-Val-L-Phe-Z-ΔPhe-OH	-6.5	-7.3	-5.7

3. *Critical Aggregation Concentration*

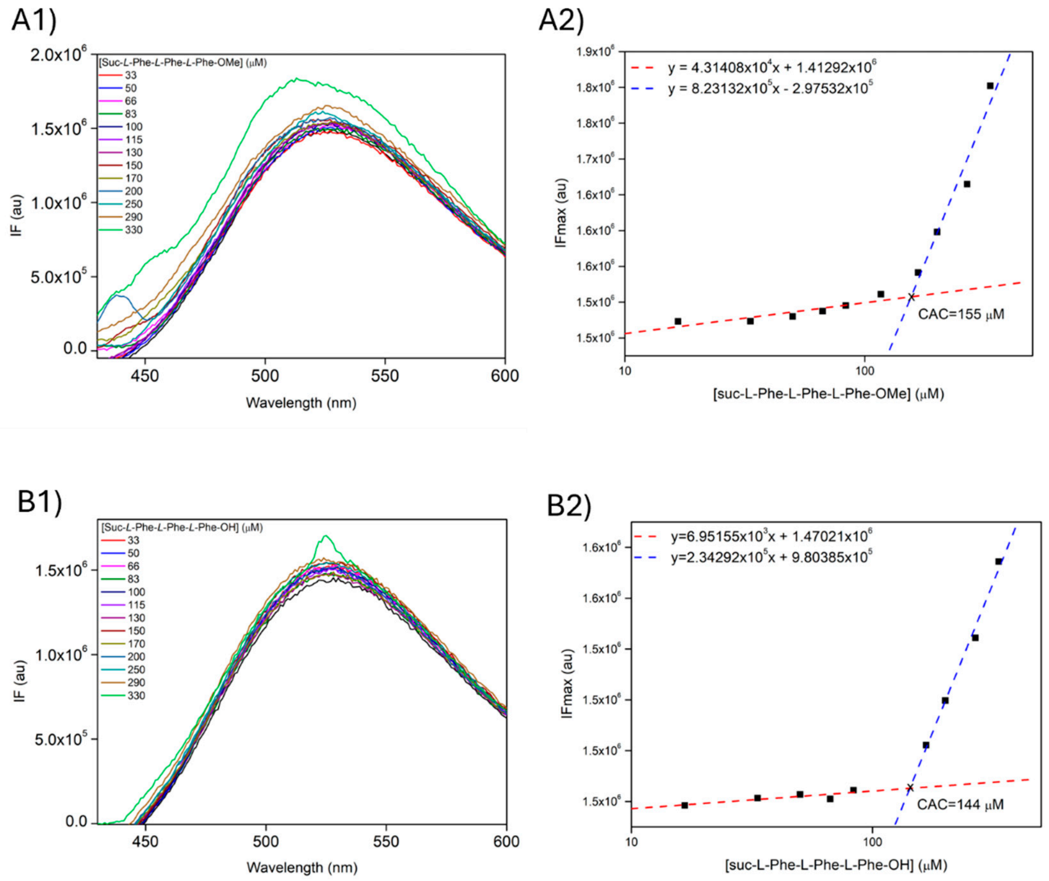

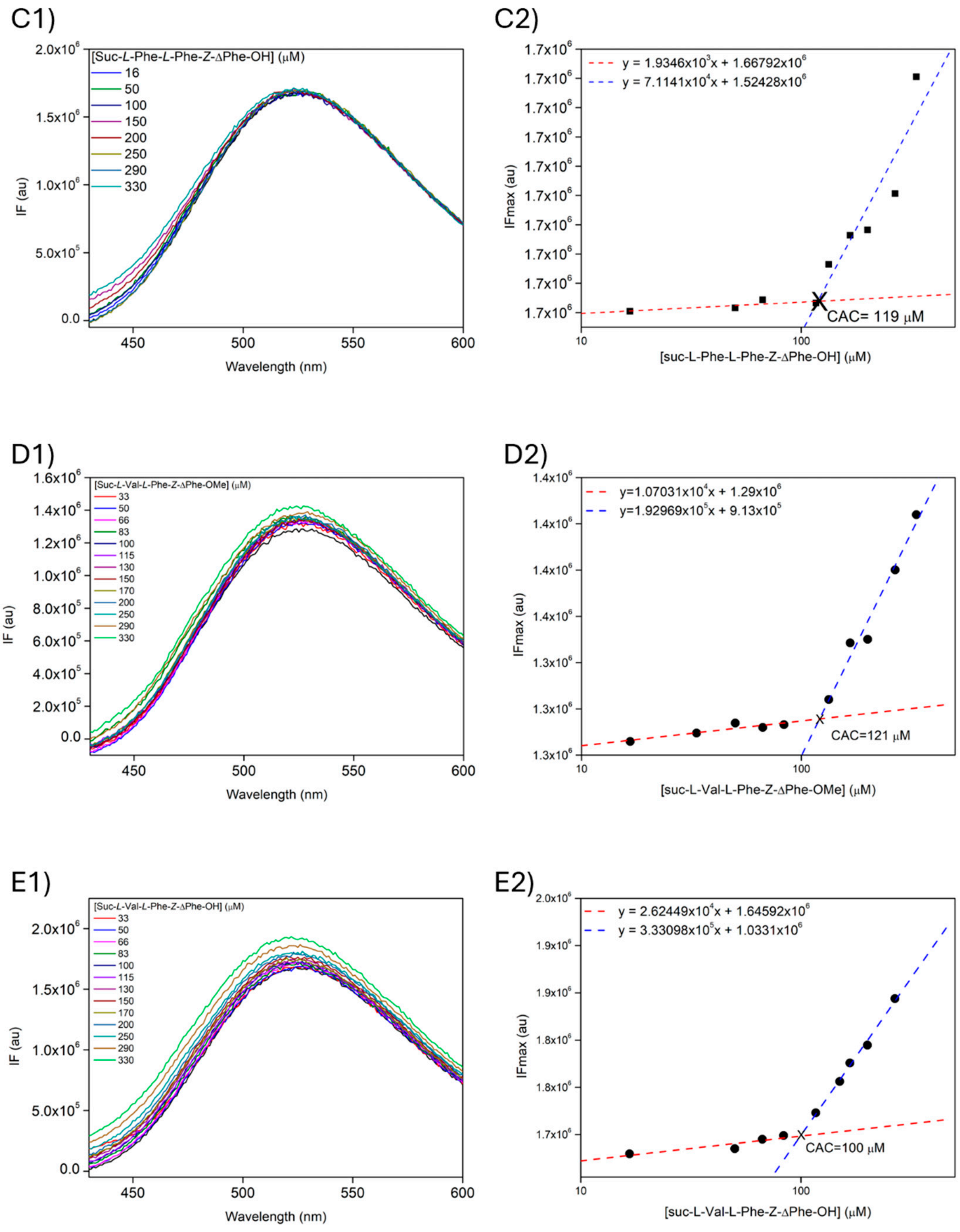

Figure S2. Experimental methodology for determination of the *CAC* values for the (dehydro)peptides: 1) Steady-state fluorescence spectra ($\lambda_{ex c}$= 370 nm) in the concentration range 330 to 16 μM in the presence of added ANS (25 μM); 2) semilogarithmic graphical representation of the concentration dependence of the fluorescence maximum emission intensity of the ANS probe (λ_{ex}= 370 nm). A) Suc-L-Phe-L-Phe-L-Phe-OMe (**10**); B) Suc-L-Phe-L-Phe-L-Phe-OH (**11**); C) Suc-L-Phe-L-Phe-Z-ΔPhe-OH (**9**); D) Suc-L-Val-L-Phe-Z-ΔPhe-OMe (**6**); E) Suc-L-Val-L-Phe-Z-ΔPhe-OH (**8**).

Table S2. (Dehydro)peptides´solubility.

(Dehydro)peptide	*S*	
	mg/mL	μM
Suc-L-Phe-L-Phe-L-Phe-OMe (**10**)	0.305	532.3
Suc-L-Phe-L-Phe-L-Phe-OH (**11**)	0.392	700.3
Suc-L-Phe-L-Phe-Z-ΔPhe-OMe (**7**)	0.197	352.7
Suc-L-Phe-L-Phe-Z-ΔPhe-OH (**9**)	0.321	561.4
Suc-L-Val-L-Phe-Z-ΔPhe-OMe (**6**)	0.296	565.7
Suc-L-Val-L-Phe-Z-ΔPhe-OH (**8**)	0.316	620.9

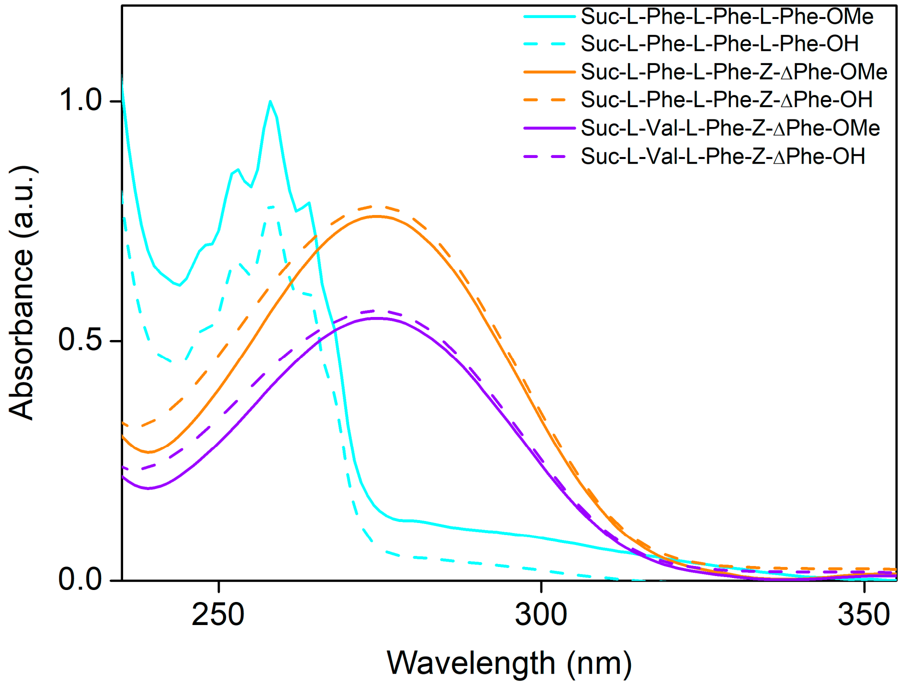

Figure S3. UV-VIS spectra of the (dehydro)peptides (300 μM, PBS pH7.4)

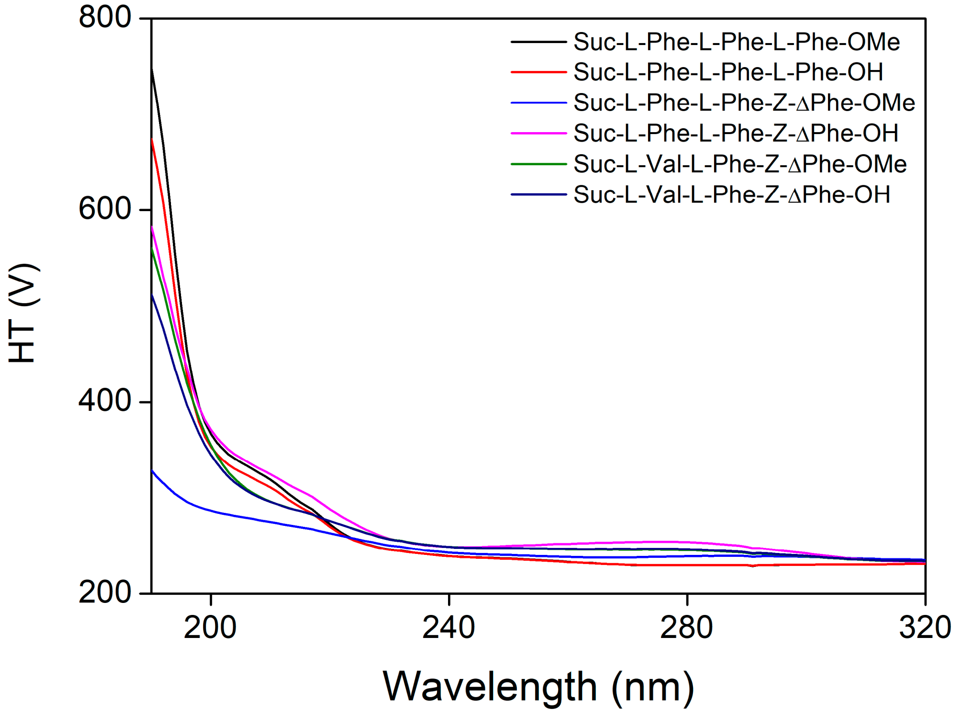

Figure S4. High Tension (HT) spectra corresponding to the CD spectra shown in Figure 4 in the manuscript.

4. *Molecular Dynamic* Simulations

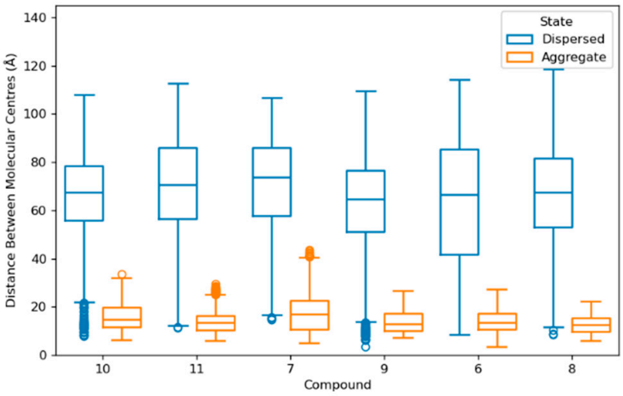

Figure S5. Box plot showing the distribution of the distance between the molecular centres for the (dehydro)peptides in the Dispersed (D) and Aggregated (Cluster)(A) states: **10**- Suc-L-Phe-L-Phe-L-Phe-OMe; **11**- Suc-L-Phe-L-Phe-L-Phe-OH; **7**- Suc-L-Phe-L-Phe-Z-ΔPhe-OMe; **9**- Suc-L-Phe-L-Phe-Z-ΔPhe-OH; **6**- Suc-L-Val-L-Phe-Z-ΔPhe-OMe; **8**- Suc-L-Val-L-Phe-Z-ΔPhe-OH.

Table S3. Solvent Accessible Surface Area (SASA, given as average ± standard deviation) for the dehydropeptides in the Dispersed and Aggregate states.

Compound	SASA (nm^2)		
	Dispersed	Aggregate	ΔSASA (mm^2)
**Suc-L-Phe-L-Phe-L-Phe-OMe**	1.563 ± 0.016	1.295 ± 0.009	0.268±0.018
**Suc-L-Phe-L-Phe-L-Phe-OH**	1.569 ± 0.010	1.288 ± 0.009	0.281± 0.013
**Suc-L-Phe-L-Phe-Z-ΔPhe-OMe**	1.597 ± 0.027	1.306 ± 0.010	0.291±0.029
**Suc-L-Phe-L-Phe-Z-ΔPhe-OH**	1.576 ± 0.013	1.281 ± 0.008	0.295±0.015
**Suc-L-Val-L-Phe-Z-ΔPhe-OMe**	1.476 ± 0.011	1.283 ± 0.009	0.193±0.014
**Suc-L-Val-L-Phe-Z-ΔPhe-OH**	1.545 ± 0.015	1.277 ± 0.009	0.268±0.018

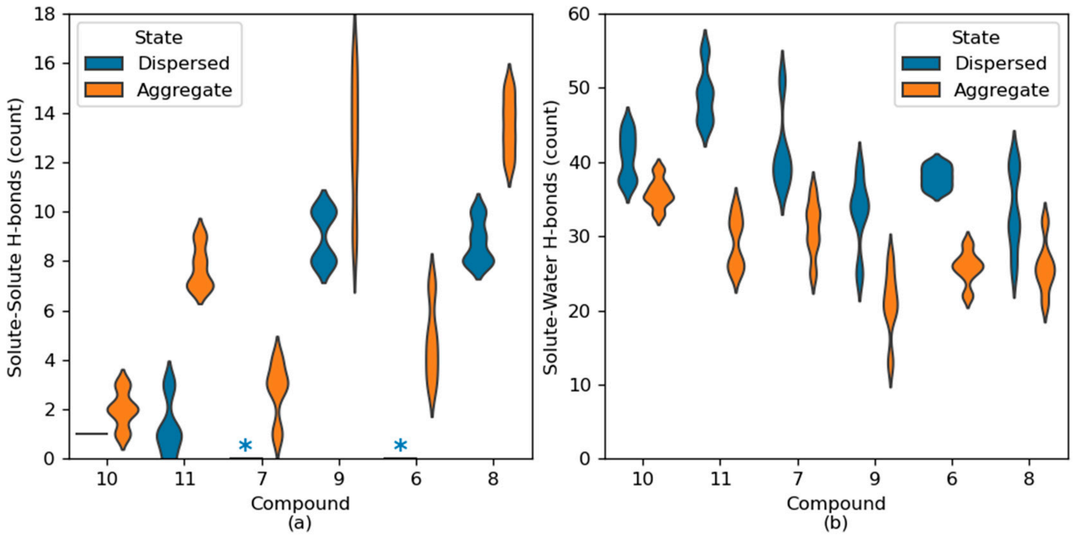

Figure S6. Distribution of peptide-peptide (A) and peptide-water (B) hydrogen bond counting in the production MD simulations for the studied dehydropeptides in the Dispersed and Aggregate state: **10**- Suc-L-Phe-L-Phe-L-Phe-OMe; **11**- Suc-L-Phe-L-Phe-L-Phe-OH; **7**- Suc-L-Phe-L-Phe-Z-ΔPhe-OMe; **9**- Suc-L-Phe-L-Phe-Z-ΔPhe-OH; **6**- Suc-L-Val-L-Phe-Z-ΔPhe-OMe; **8**- Suc-L-Val-L-Phe-Z-ΔPhe-OH.

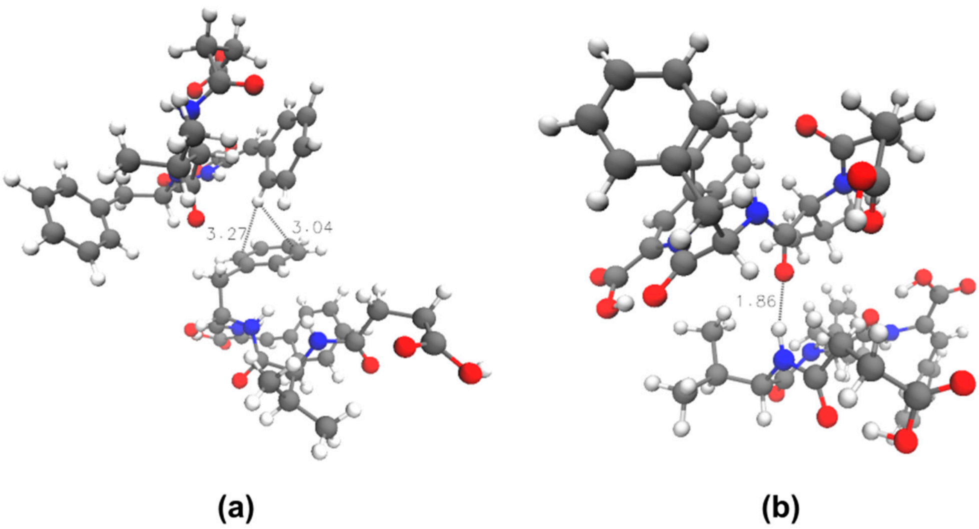

Figure S7. Depiction of molecular pairs of dehydropeptide Suc-L-Val-L-Phe-Z-ΔPhe-OH (**8**) in the aggregate phase during the MD simulations, exemplifying: a) the approximate geometry of the T-shaped π-π interaction between the aromatic rings of Phe; b) intermolecular hydrogen bond between the NH of Val and the C=O of the Val residue of another molecule.

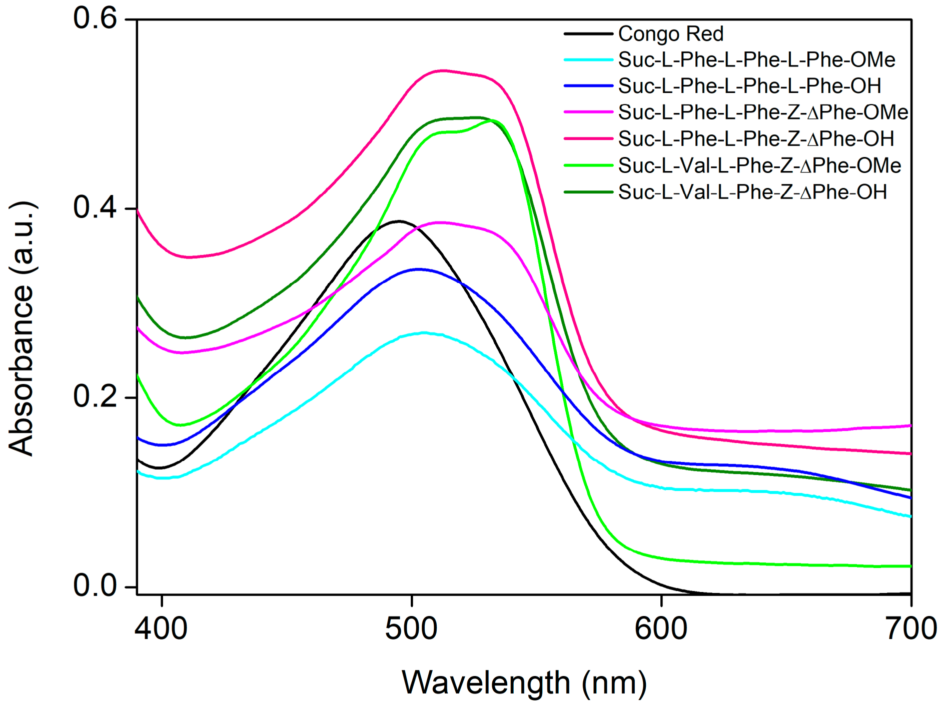

Figure S8. UV-Vis absorption spectra of Congo red in PBS in the absence (black trace) and presence (colored traces) of peptide hydrogel fibers.

5. *ATR FTIR Deconvolution*

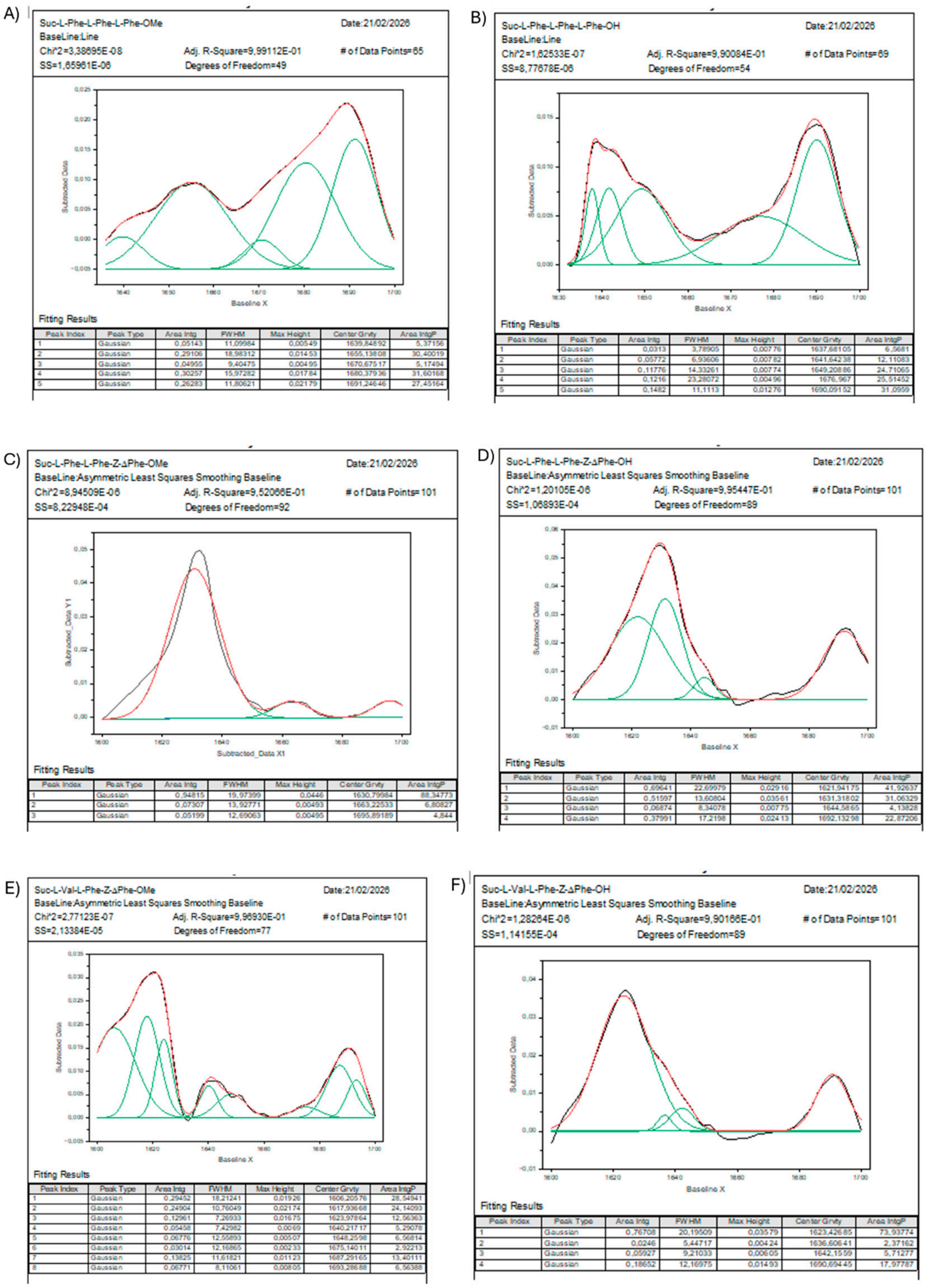

Figure S9. The deconvolution of the Amide I region (1700-1600 cm^{-1}) of the FTIR spectra was accomplished using OriginPro software. Baseline correction was performed using the ALS protocol. The initial peak positions were determined from the second-derivative of the spectrum. The experimental curve (black line) was fitted by nonlinear least-squares analysis using Gaussian functions to resolve overlapping component bands (green curves). The red curve represents the cumulative fit (sum of all Gaussian components), demonstrating good agreement with the experimental data. Peak positions, full width at half maximum (FWHM), and intensities were

iteratively optimized until convergence was achieved minimizing the residual error. The resolved sub-bands are assigned to different protein secondary structure contributions within the Amide I envelope. A) Suc-L-Phe-L-Phe-L-Phe-OMe (**10**); B) Suc-L-Phe-L-Phe-L-Phe-OH (**11**); C) Suc-L-Phe-L-Phe-Z-ΔPhe-OMe (**7**); D) Suc-L-Phe-L-Phe-Z-ΔPhe-OH (**9**); E) Suc-L-Val-L-Phe-Z-ΔPhe-OMe (**6**); F) Suc-L-Val-L-Phe-Z-ΔPhe-OH (**8**).

Table S4. Deconvolution of the amide I region of the ATR FTIR spectra

**Hydrogel**	**Peak position (cm^{-1})<br>Relative area (%)**							
Suc-L-Phe-L-Phe-L-Phe-OMe (**10**)	1640<br>5%	1655<br>31%	1671<br>5%	1680<br>32%	1691<br>27%	-	-	-
Suc-L-Phe-L-Phe-L-Phe-OH (**11**)	1638<br>6%	1642<br>12%	1649<br>25%	1677<br>26%	1690<br>31	-	-	-
Suc-L-Phe-L-Phe-Z-ΔPhe-OMe (**7**)	1631<br>88%	1663<br>7%	1695<br>5%	-	-	-	-	-
Suc-L-Phe-L-Phe-Z-ΔPhe-OH (**9**)	1622<br>42%	1631<br>31%	1645<br>4%	1692<br>23%	-	-	-	-
Suc-L-Val-L-Phe-Z-ΔPhe-OMe (**6**)	1606<br>29%	1618<br>24%	1624<br>13%	1640<br>5%	1648<br>7%	1675<br>3%	1687<br>13%	1693<br>6%
Suc-L-Val-L-Phe-Z-ΔPhe-OH (**8**)	1623<br>74%	1637<br>2%	1642<br>6%	1690<br>18%	-	-	-	-

6. *Rheological Characterization*

6.1. Suc-L-Phe-L-Phe-L-Phe-OMe

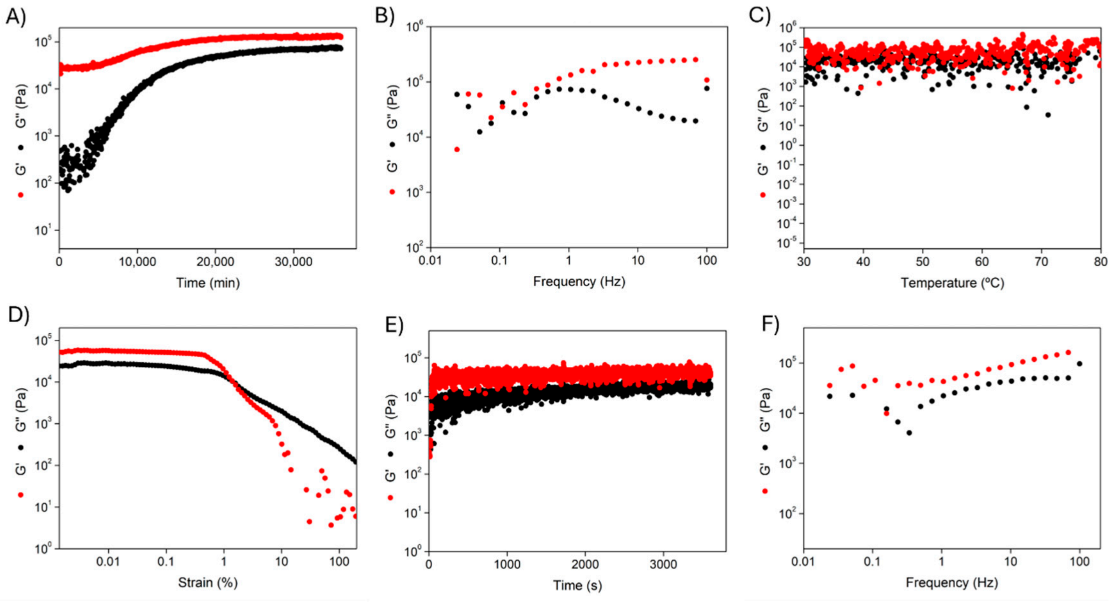

6.2. Suc-L-Phe-L-Phe-L-Phe-OH

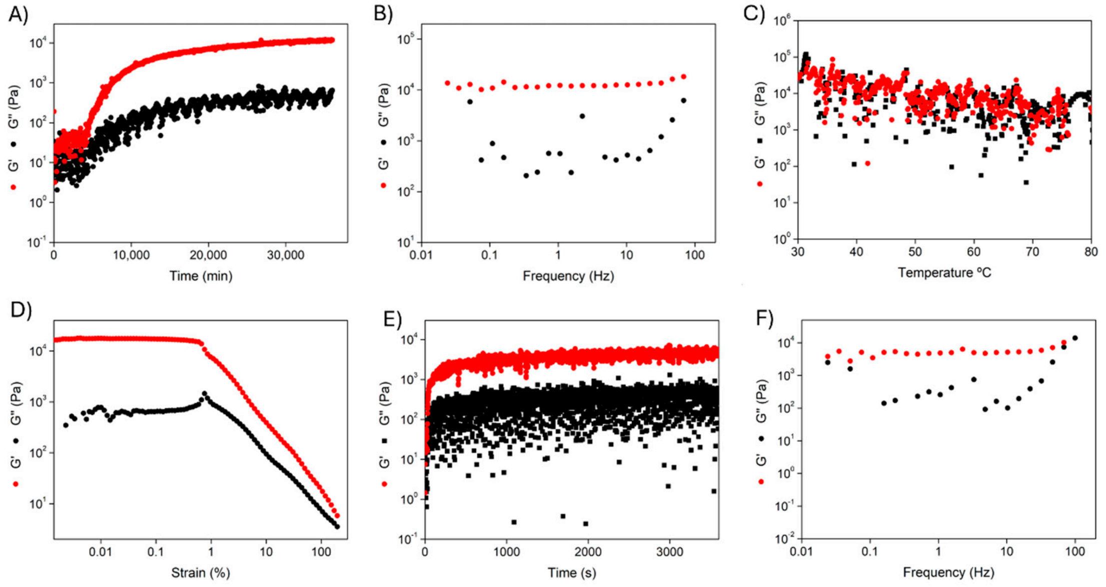

6.3. Suc-L-Phe-L-Phe-Z-ΔPhe-OH

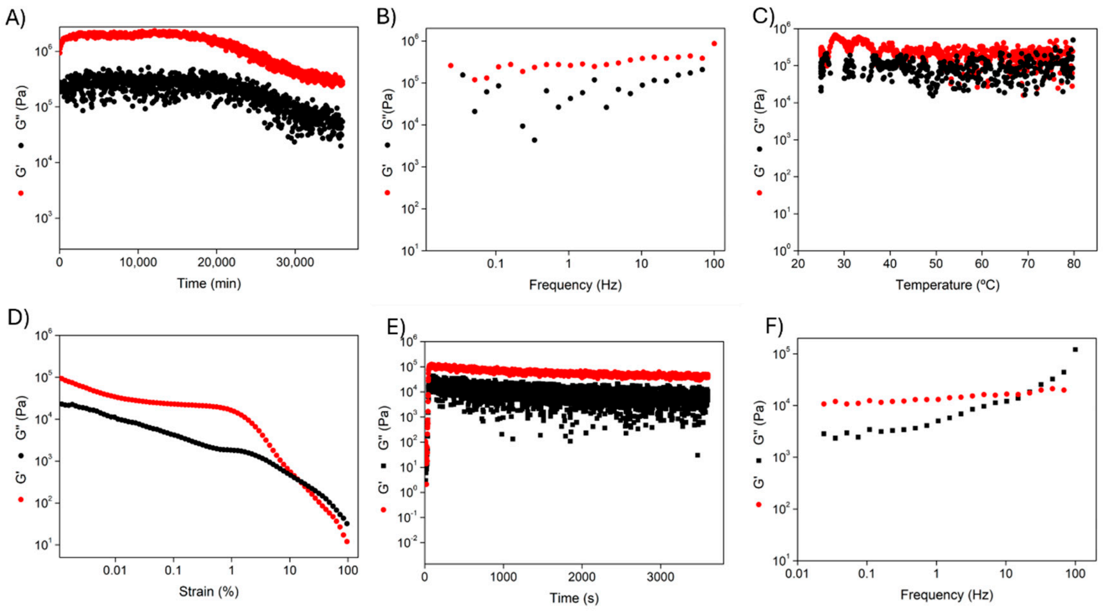

6.4. Suc-L-Val-L-Phe-Z-ΔPhe-OMe

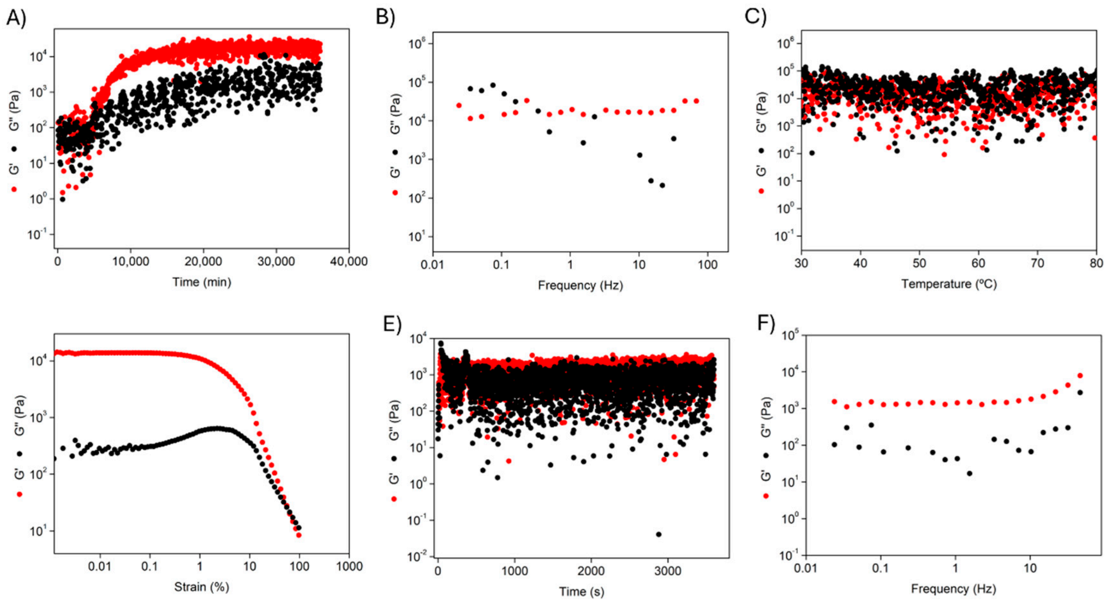

6.5. Suc-L-Val-L-Phe-Z-ΔPhe-OH

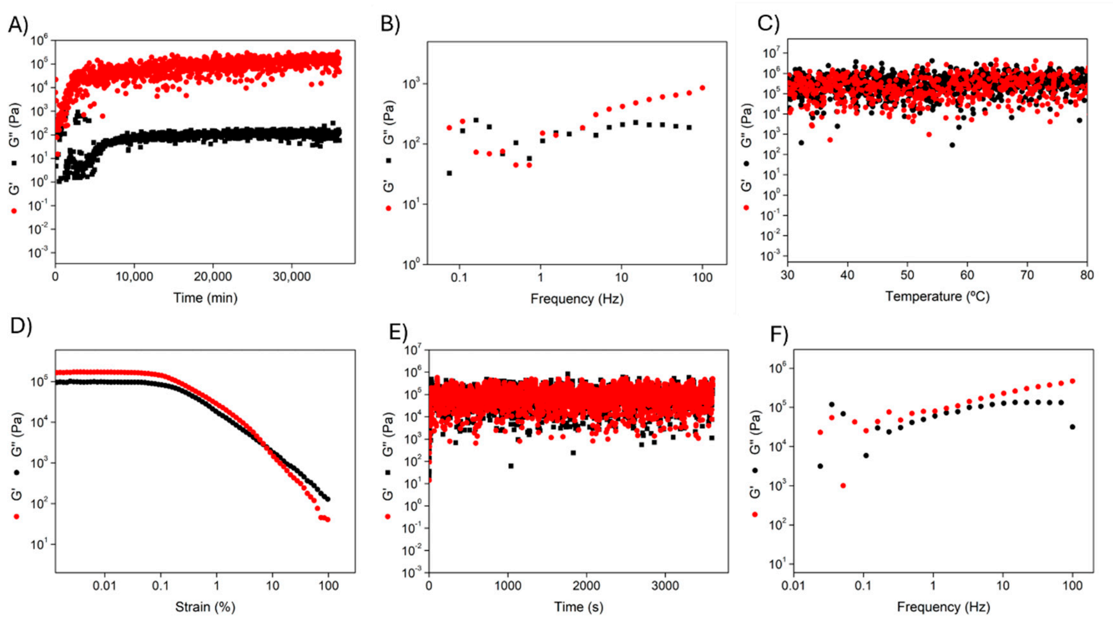

Figure S10. Detailed experimental protocol implemented for the characterization of (dehydro)tripeptide hydrogels: A) gelation kinetics; B) Mechanical spectrum, frequency sweep, for equilibrated gels; C) Temperature cycling; D) Strain sweep on *rested gels* after temperature cycling; E) Hydrogel reformation kinetics after mechanical breakdown; F) Mechanical spectrum, frequency sweep, for reformed hydrogels. 6.1) Suc-L-Phe-L-Phe-L-Phe-OMe (**10**); 6.2) Suc-L-Phe-L-Phe-L-Phe-OH (**11**); 6.3) Suc-L-Phe-L-Phe-Z-ΔPhe-OH (**9**); 6.4) Suc-L-Val-L-Phe-Z-ΔPhe-OMe (**6**); 6.5) Suc-L-Val-L-Phe-Z-ΔPhe-OH (**8**).

7. *Drug-release*

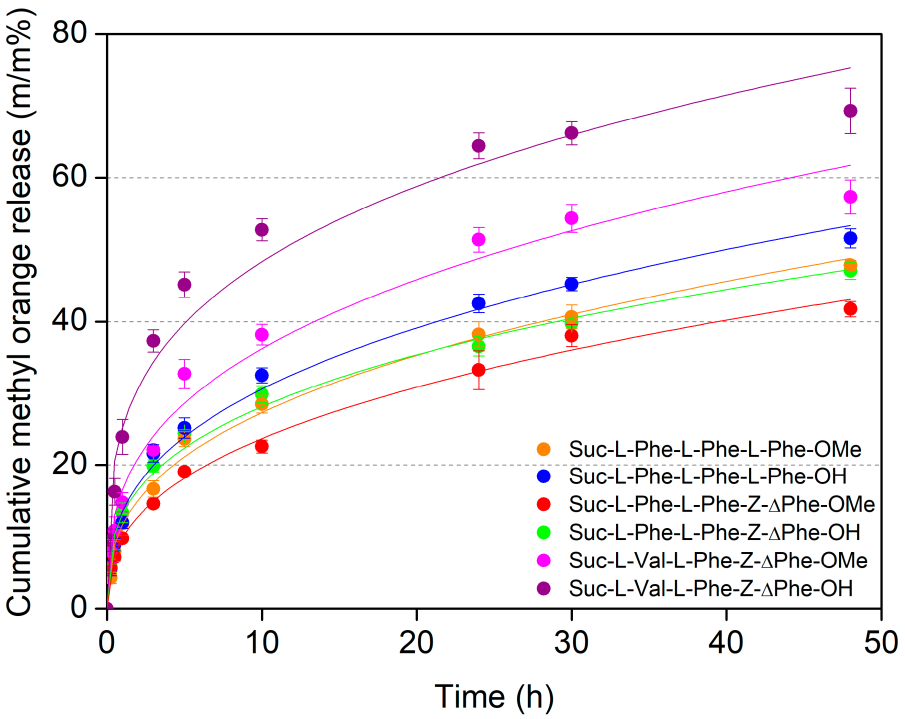

Figure S11. Methyl Orange-release profiles for (dehydro)peptide hydrogels fitted to the Korsmeyer-Peppas model (solid lines).

Table S5. Best parameters for the fitting of the experimental Methyl Orange-release profiles of the (dehydro)peptide hydrogels to the *Weibull* and *Korsmeyer-Peppas* models.

**Hydrogel**	*Korsmeyer-Peppas*				*Weibull*				
	K	***n***	***R²***	***RMSE***	*M∞*	***d***	***k***	***R²***	***RMSE***
Suc-L-Phe-L-Phe-L-Phe-OMe (**10**)	11.7±0.64	0.40±0.02	0.992	1.499	63.9±7.2	0.48±0.04	0.04±0,02	0.994	1.163
Suc-L-Phe-L-Phe-L-Phe-OH (**11**)	13.5±0.74	0.35±0.02	0.990	1.723	67.9±10.8	0.47±0,05	0.04±0,02	0.996	0.737
Suc-L-Phe-L-Phe-Z-ΔPhe-OMe (**7**)	9.8±0.43	0.38±0.02	0.995	1.011	69.4±10.7	0.47±0,03	0.02±0,01	0.994	1.096
Suc-L-Phe-L-Phe-Z-ΔPhe-OH (**9**)	13.1±0.7	0.33±0.02	0.990	1.601	56.5±9.8	0.49±0,07	0.05±0,04	0.996	1.373
Suc-L-Val-L-Phe-Z-ΔPhe-OMe (**6**)	16.6±1.3	0.34±0.02	0.980	2.895	65.3±5.8	0.60±0,08	0.09±0,03	0.993	1.579
Suc-L-Val-L-Phe-Z-ΔPhe-OH (**6**)	25.2±2.1	0.28±0.03	0.965	4.708	72.7±4.8	0.50±0,06	0.17±0,05	0.997	1.179

8. Enzymatic Hydrolyses

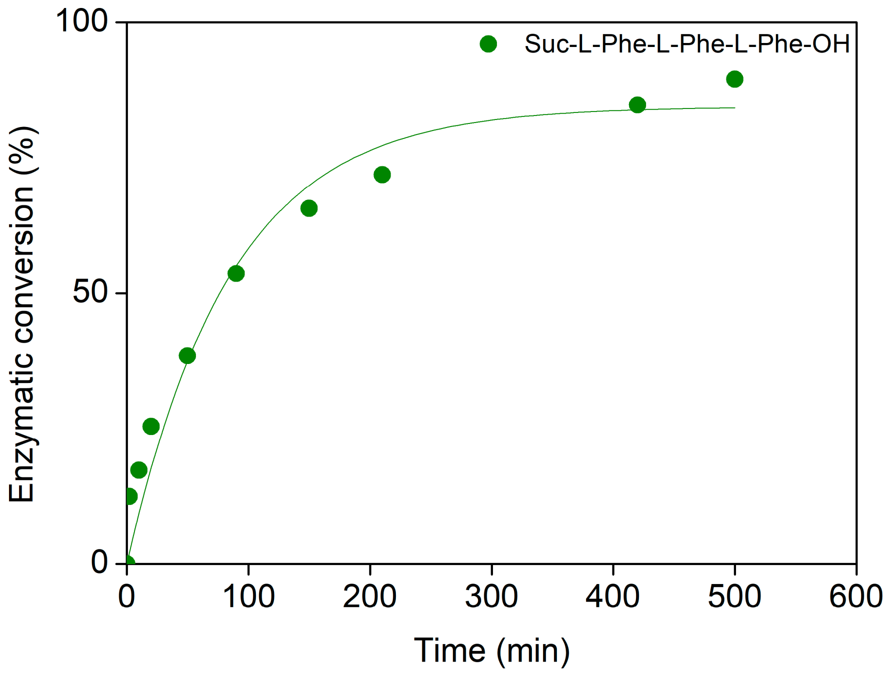

Figure S12. Conversation degree for chymotrypsin-catalyzed hydrolysis of the canonical dicarboxylic acid Suc-L-Phe-L-Phe-L-Phe-OH (**11**). The time evolution of the hydrolysis was fitted to a pseudo first order model (solid line).

Table S6. Best-fit parameters for the chymotrypsin-catalyzed hydrolysis of canonical dicarboxylic acid Suc-L-Phe-L-Phe-L-Phe-OH (**11**) to a pseudo first order model (equation 2 in the main text).

Hydrogelator	$C\infty$	k	R^2
Suc-L-Phe-L-Phe-L-Phe-OH (**11**)	84.45	0.01	0.961

Table S7. Best-fit parameters for the fitting of the experimental conversation rate for the chymotrypsin-catalysed methyl ester hydrolysis to a pseudo first order model (equation 2 in the main text).

Hydrogelator	$C\infty$	k	R^2
Suc-L-Phe-L-Phe-L-Phe-OMe (**10**)	99.61	0.91	0.999
Suc-L-Phe-L-Phe-Z-ΔPhe-OMe (**7**)	97.42	0.03	0.977
Suc-L-Val-L-Phe-Z-ΔPhe-OMe (**6**)	92.36	0.20	0.943

9. *Enzymatic Hydrolysis*

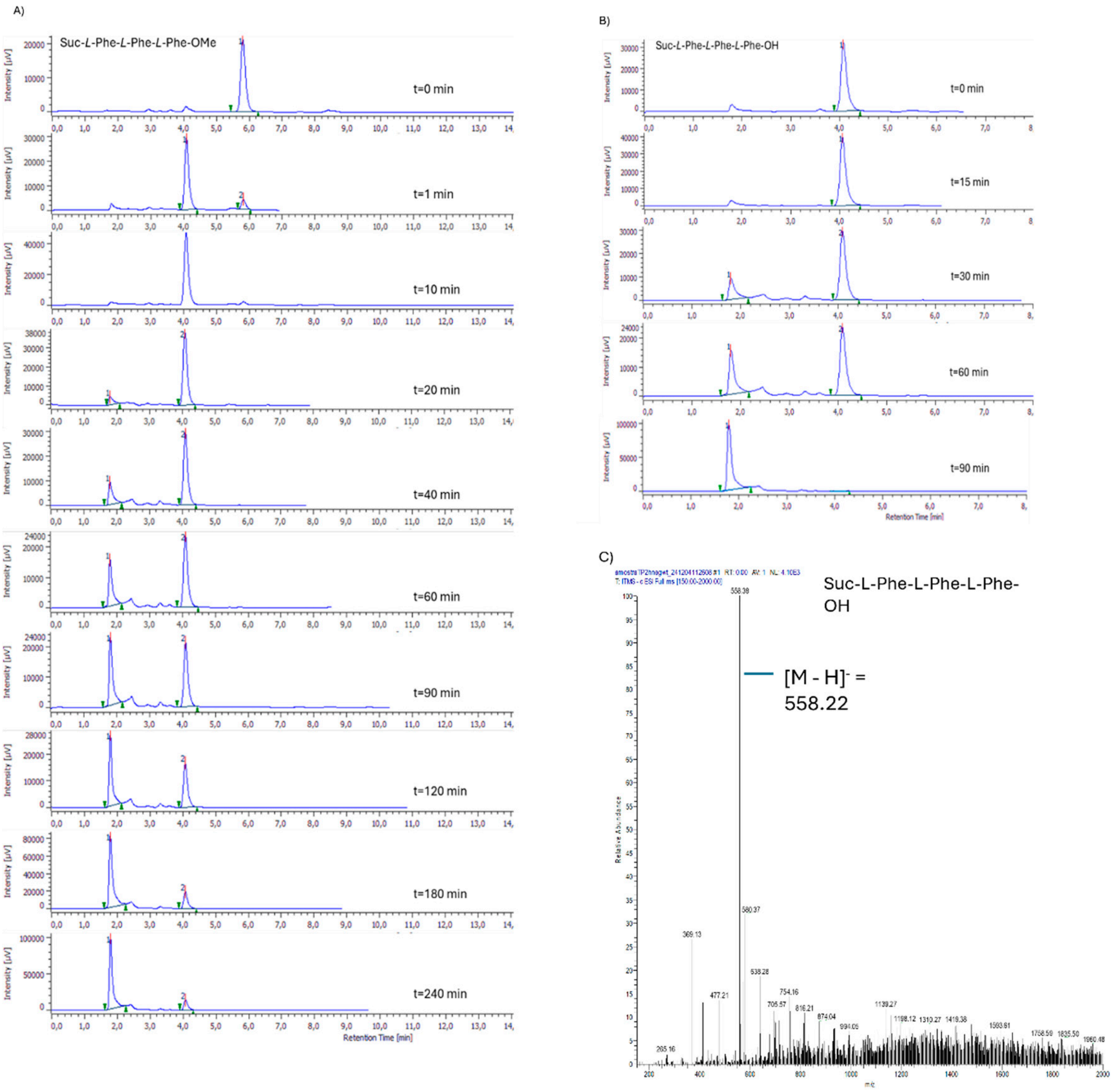

Figure S13. RP HPLC profiles for chymotrypsin-catalysed hydrolysis: A) Suc-L-Phe-L-Phe-L-Phe-OMe (**10**); B) Suc-L-Phe-L-Phe-L-Phe-OH (**11**). C) MS spectrum of the hydrolysis product of ester Suc-L-Phe-L-Phe-L-Phe-OMe (**11**).

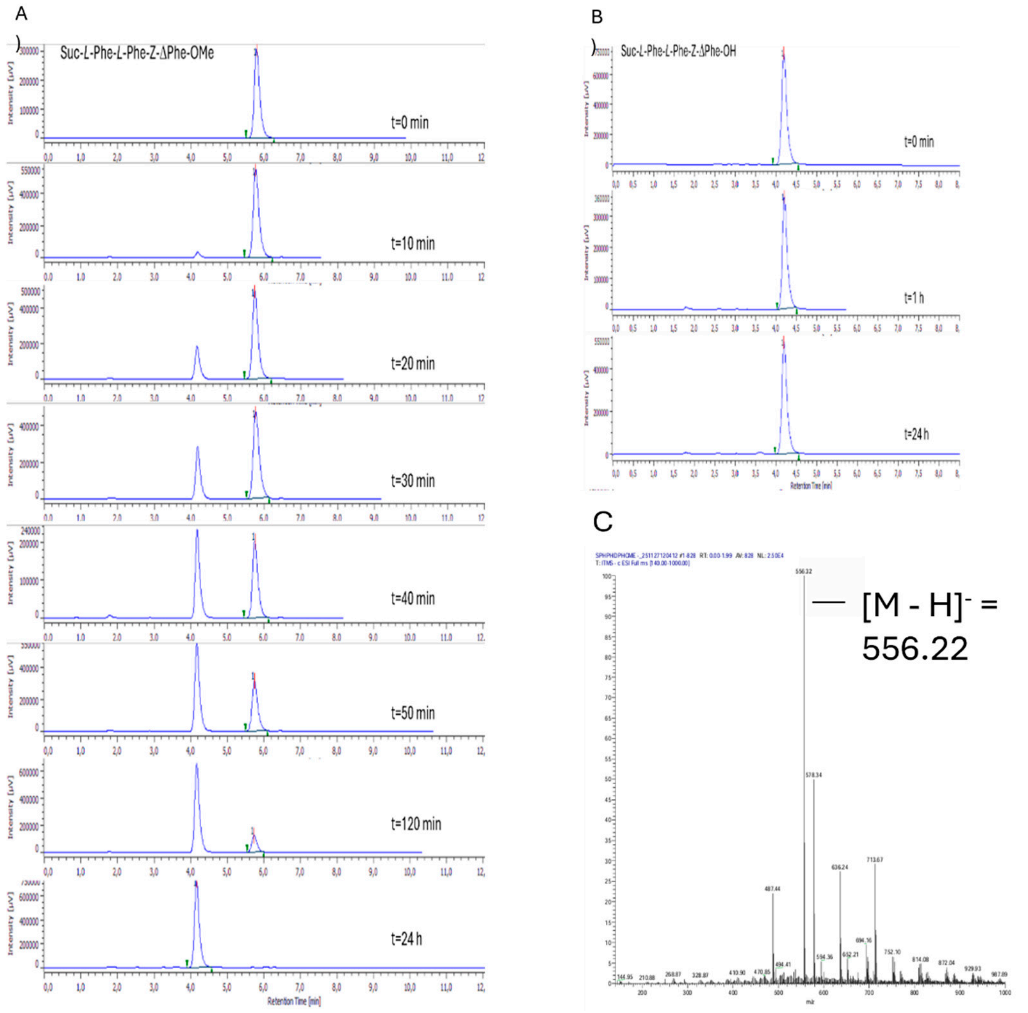

Figure S14. HPLC profiles for chymotrypsin-catalysed hydrolysis: A) Suc-L-Phe-L-Phe-Z-ΔPhe-OMe (**7**) and B) Suc-L-Phe-L-Phe-Z-ΔPhe-OH (**9**). C) MS spectrum of the hydrolysis product of ester Suc-L-Phe-L-Phe-Z-ΔPhe-OMe.

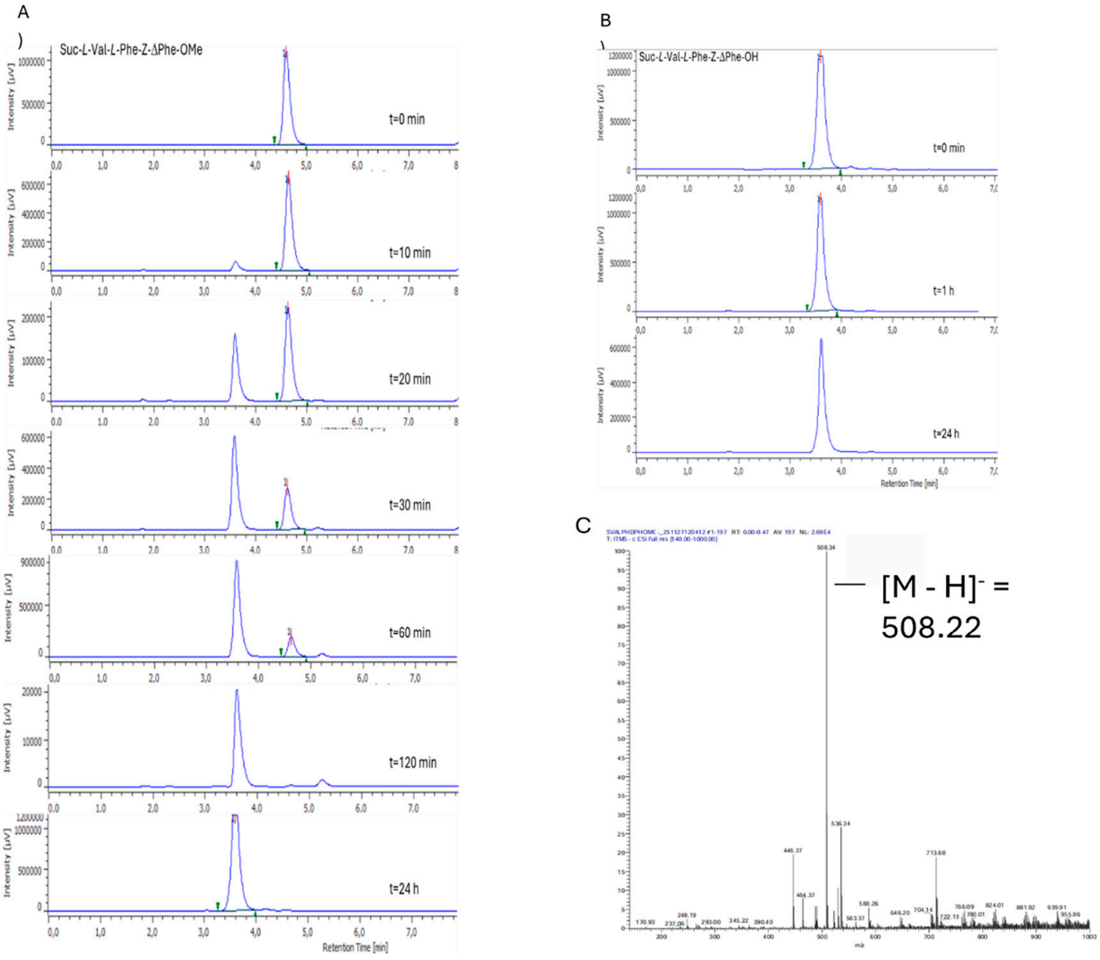

Figure S15. HPLC profiles for chymotrypsin-catalysed hydrolysis: A) Suc-L-Val-L-Phe-Z-ΔPhe-OMe (**6**) and B) Suc-L-Val-L-Phe-Z-ΔPhe-OH (**8**). C) MS spectrum of the hydrolysis product of ester Suc-L-Val-L-Phe-Z-ΔPhe-OMe (**6**).
